# Clinical significance of nonerythrocytic spectrin Beta 1 (SPTBN1) in human kidney renal clear cell carcinoma and uveal melanoma: a study based on Pan-Cancer Analysis

**DOI:** 10.1186/s12885-023-10789-3

**Published:** 2023-04-03

**Authors:** Wenting Tang, Qiong Shao, Zhanwen He, Xu Zhang, Xiaojuan Li, Ruohao Wu

**Affiliations:** 1 State Key Laboratory of Oncology in South China, Collaborative Innovation Center for Cancer Medicine, Sun Yat-sen University Cancer Center, Sun Yat-sen University, Guangzhou, 510060 Guangdong China; 2 Department of Research and Molecular Diagnostics, Sun Yat-sen University Cancer Center, Sun Yat-sen University, Guangzhou, 510060 Guangdong China; 3 Guangdong Provincial Key Laboratory of Malignant Tumor Epigenetics and Gene Regulation, Sun Yat-sen Memorial Hospital, Sun Yat-sen University, Guangzhou, 510120 Guangdong China; 4 Department of Pediatrics, Sun Yat-sen Memorial Hospital, Sun Yat-sen University, Guangzhou, 510120 Guangdong China; 5 Department of Research and Molecular Diagnostics, Sun Yat-sen Memorial Hospital, Sun Yat-sen University, Guangzhou, 510120 Guangdong China

**Keywords:** Nonerythrocytic spectrin beta 1, Pan-cancer, Prognosis, Tumor immunity, KIRC, UVM

## Abstract

**Background:**

Nonerythrocytic spectrin beta 1 (SPTBN1) is an important cytoskeletal protein that involves in normal cell growth and development via regulating TGFβ/Smad signaling pathway, and is aberrantly expressed in various cancer types. But, the exact role of SPTBN1 in pan-cancer is still unclear. This report aimed to display expression patterns and prognostic landscapes of SPTBN1 in human cancers, and further assess its prognostic/therapeutic value and immunological role in kidney renal carcinoma (KIRC) and uveal melanoma (UVM).

**Methods:**

We firstly analyzed expression patterns and prognostic landscapes of SPTBN1 in human cancers using various databases and web-based tools. The relationships between SPTBN1 expression and survival/tumor immunity in KIRC and UVM were further investigated via R packages and TIMER 2.0 platform. The therapeutic roles of SPTBN1 in KIRC and UVM were also explored via R software. Following this, the prognostic value and cancer immunological role of SPTBN1 in KIRC and UVM were validated in our cancer patients and GEO database.

**Results:**

Overall, cancer tissue had a lower expression level of SPTBN1 frequently in pan-cancer, compared with those in adjacent nontumor one. SPTBN1 expression often showed a different effect on survival in pan-cancer; upregulation of SPTBN1 was protective to the survival of KIRC individuals, which was contrary from what was found in UVM patients. In KIRC, there were significant negative associations between SPTBN1 expression and pro-tumor immune cell infiltration, including Treg cell, Th2 cell, monocyte and M2-macrophage, and expression of immune modulator genes, such as tumor necrosis factor superfamily member 9 (TNFSF9); while, in UVM, these correlations exhibited opposite patterns. The following survival and expression correlation analysis in our cancer cohorts and GEO database confirmed these previous findings. Moreover, we also found that SPTBN1 was potentially involved in the resistance of immunotherapy in KIRC, and the enhance of anti-cancer targeted treatment in UVM.

**Conclusions:**

The current study presented compelling evidence that SPTBN1 might be a novel prognostic and therapy-related biomarker in KIRC and UVM, shedding new light on anti-cancer strategy.

**Supplementary Information:**

The online version contains supplementary material available at 10.1186/s12885-023-10789-3.

## Introduction

Human cancer is a very complicated disorder and tumorigenesis is closely correlated with the disturbance of genetics and immune system[1]. Therefore, it is meaningful to initiate a pan-cancer study of any tumor-involved gene and explore its correlations with prognosis and cancer immunity. Thanks to the available of various public databases with huge transcript abundance and many powerful integrated web-based bioinformatic tools for human cancer, such as the cancer genome atlas (TCGA) project[2], clinical proteomic analysis consortium (CPTAC) dataset[3], gene expression omnibus (GEO) database[4], tumor immune estimation resource (V.2.0) (TIMER2.0) platform[5] and University of ALabama at Birmingham CANcer (UALCAN) data analysis portal[6], we could perform comprehensive pan-cancer studies of any gene easily.

Spectrin, as an important cytoskeletal protein involving in maintaining cell structure and membrane stability, was first discovered in red cells in 1968[7, 8]. After that, many different spectrin isoforms were successively discovered. Based on their tissue/cell distribution, spectrin protein could be classified into two types: erythroid spectrin isoforms (α-spectrin isoforms) and nonerythroid spectrin isoforms (β-spectrin isoforms). Among these nonerythroid spectrin isoforms, nonerythrocytic spectrin beta 1 (SPTBN1), also terms as βII-spectrin, is one of conventional β-spectrin isoforms being encoded by *SPTBN1* gene and has been revealed to be necessary for maintaining normal function or cell shape of epithelials[9]. Moreover, SPTBN1 also has multiple biological functions, including cytoskeleton construction, cell motion and adhesion, and ion transport[10]. More importantly, SPTBN1, acting as an essential regulator of Smad3/4 complex, can regulate of transforming growth factor-β (TGF-β) signaling pathway, maintaining normal cell growth, cell development and cell differentiation[11–13]. Several studies of embryonic lethality in SPTBN1^−/−^ mice demonstrated that pathogenic variants in *SPTBN1* could cause severe deficits of multiple organs, including brain, cardiovascular system, intestine and liver[14–16]. Recently, SPTBN1 is found to be related to many types of human cancers, such as lymphoma, lung carcinoma, esophageal carcinoma, liver cancer, ovarian tumor and colorectal carcinoma[17–20]. Some studies revealed that SPTBN1 could contribute to anti-cancer at early stage of cancer via regenerating and repairing damaged tissue[21–23]; while, other publications demonstrated that SPTBN1 played important roles in cancer development and tumor metastasis via promoting the process of epithelial mesenchymal transition (EMT)[24, 25]. Relevance of SPTBN1 biological function and molecular mechanism in pan-cancer have been largely underexplored and the role of SPTBN1 in cancer development could be paradoxical and might vary among different types or stages/grades of cancers[20]. For instance, decreased SPTBN1 expression could promote tumorigenesis and malignant manners, and act as a poor prognosis factor in some human cancers, like hepatocellular carcinoma[26] or high grade of pancreatic cancer[27]; however, increased expression of SPTBN1 could also promote carcinogenesis and predict poor prognosis in some cancers, such as ovarian cancer[28] and stage IV of colon cancer[29]. Thus, it still remains unclear whether SPTBN1 could be regarded as a friend or woe in pan-cancer.

Previous researches demonstrated that tumor immunity and tumor microenvironment (TME) acted vital roles in cancer development and treatment[30]. TME includes varieties of cells, such as tumor immune infiltrating cells (TIICs) and stromal cell[30]. Among these cells, regulatory T cell (Treg), helper T cell type 2 (Th1), monocyte, M2-macrophage, endothelial cell and cancer-associated fibroblast are the primary effector TIICs and stromal immune cells in cancer immunity. For examples, M2-macrophage and Treg could contribute to tumor progression by helping cancer cells escape and/or promoting EMT and abnormal angiogenesis[30]. In addition, cancer-associated endothelial cell and fibroblast could also participate in development of tumor invasion and metastasis[30]. In contrast, immunotherapy targeting specific immune checkpoints related to immune escape has been developed rapidly and becomes a robust alternative anti-tumor strategy to classic anti-cancer therapies[31]. For examples, tumor necrosis factor superfamily member 9 (TNFSF9), programmed cell death-1 (PDCD1) and cytotoxic T-lymphocyte-associated protein-4 (CTLA4) are the common immune checkpoint markers in tumor immunotherapy and have been maturely applied in treatments of many kinds of cancers, including kidney carcinoma[32] and malignant uveal melanoma[33]. Moreover, TGF-β1 is an important known dual-immunomodulatory factor in cancer and plays an important role in affecting the infiltration of multiple types of TIICs and the expression of immune checkpoint genes[34]. By binding to the Smad3/4 complex, SPTBN1 might regulate tumor immunity or cancer progression via the TGF-β/Smad signaling pathway[35]. Hence, SPTBN1 is a potential candidate immunomodulatory factor and immunotherapy target in cancer. Exploiting exact roles of SPTBN1 in human cancer progression will contribute to tumor prognosis and treatment. However, the correlation between function of SPTBN1 and cancer prognosis or tumor immunity remains unclear.

In current report, we explore expression patterns of SPTBN1 in pan-cancer, and visualize its prognostic landscape in human cancers, especially in kidney renal carcinoma (KIRC) and malignant uveal melanoma (UVM). Then, we explored the potential correlations between SPTBN1 expression and TIICs/immune modulator markers/response of immunotherapy or anti-cancer targeted treatment in KIRC and UVM. The findings from current report demonstrated for the first time that when facing different types or grades/stages of human cancers, such as KIRC and UVM, SPTBN1 could serve as a dual-marker for predicting cancer prognosis and response of anti-cancer therapy.

## Materials and methods

### Expression patterns of SPTBN1 in human cancers

The analysis of differential expression of SPTBN1 at mRNA and protein levels between tumor and corresponding adjacent nontumor tissues were based on the cancer genome atlas (TCGA) database[2] and clinical proteomic tumor analysis consortium (CPTAC) dataset[3], respectively. Specifically, the analysis of these mRNA expression data in pan-cancer was conducted by using online platform of TIMER 2.0 with normalization of log2 transcripts per kilobase million (log2TPM)[5]. The analysis of expression data for KIRC and UVM were conducted by using UALCAN online platform with normalizations of TPM for mRNA level and Z-score for protein level[6], respectively. The analysis of the relationships between SPTBN1 mRNA expression and the cancer stage/grade in KIRC and UVM were conducted using UALCAN online tool with normalizations of TPM.

### Survival study of SPTBN1 expression in human cancers

In present section, firstly, the SPTBN1 mRNA expression data with normalization of fragments per kilobase of exon per million readers (FPKM) and survival data across 33 types of TCGA cancers were downloaded from Genomic Data Commons (GDC)[36]. Then, the correlations between SPTBN1 mRNA expression and survival, including survival of overall (OS) and disease-specific (DSS), and progression-free interval (PFI), in these 33 TCGA cancers were analyzed using a Cox regression survival analysis in R environment with R package “survival“[37]. thresholds of Cox analysis were defined as a hazard ratio (HR) of 95% confidence intervals (95%CI) with a *p* value < 0.05. Finally, The correlations between SPTBN1 expression and survival, including OS, DSS and PFI, in KIRC and UVM were further analyzed using Kaplan-Meier curve with a Log-rank *p* method in R environment with R packages “survminer“[38] and “survival“[37].

### Assessment of the Relationships between SPTBN1 expression and Immune Microenvironment in KIRC and UVM

It is widely acknowledged that TME acts an important role in cancer development and prognosis[30]. Thus, in order to explore the correlations of the estimated proportions of stromal and immune components in TME with SPTBN1 expression, we firstly used the “ESTIMATE” algorithm in R environment with R packages “estimate“[39] and “limma“[40] to analyze stromal and immune cells in TME in KIRC and UVM by evaluating the scores of stromal and immune, and total ESTIMATE score of each specimen, and visualized these results in R environment with R package “ggplot2“[41]. Then, by using TIMER 2.0 online tool with a cut-off (*p*) of 0.05 and a coefficient (Spearman’s ρ) of 0[5], we further analyzed the SPTBN1 expression (log2TPM form) with the infiltration levels of 6 types of infiltrating stromal/immune cells (endothelial cell, cancer-associated fibroblast, Treg cell, Th2 cell, monocyte and M2-macrophage), which all had an important role in promoting cancer development in KIRC and UVM after adjustment of the tumor purity with SPTBN1 expression.

### Analysis of the Relationships between SPTBN1 expression and Immune Modulator marker genes in human cancers

In order to explore the relationship between SPTBN1 expression and tumor immunity comprehensively, we also analyzed and visualized the correlations between SPTBN1 expression (FPKM from) and expression levels of common immune modulator markers (FPKM from) that involved in cancer evasion[31], including TNFSF9-CD44-CD86-CD274-TIGIT-TNFSF15-TNFRSF18-CD40-TNFRSF4-VSIR-TNFRSF25-CD27-TNFRSF8-TNFSF9-CD70-BTNL2-TNFSF18-HHLA2-PDCD1LG2-IDO2-VTCN1-TIMGD2-ICOSLG-IDO2-TNFSF14-CD160-LGALS9-PDCD1-CD80-KIR3DL1-CD276-ADORA2A-HAVCR2-CD200R1-CD28-CD48-CTLA4-CD40LG-ICOS-LAG3-CD244-TNFSF4-LAIR1-NRP1-TNFRSF14-CD200-BTLA, across TCGA cancers by using R package “reshape2” [42]. In addition, for further predicting the potential immunotherapy effects of SPTBN1 in KIRC and UVM, we then explored the correlations between SPTBN1 expression (log2TPM form) and 3 common immunotherapy-related marker genes (TNFSF9, PDCD1 and CTLA4) expression (log2TPM form) in KIRC and UVM using TIMER 2.0 online tool with a cut-off (*p*) of 0.05 and a coefficient (Spearman’s ρ) of 0[5], after adjustment of the tumor purity with SPTBN1 expression.

**Validation of the Relationship between SPTBN1 and TNFSF9 Expression, and the Prognostic Role of SPTBN1 in KIRC by Immunohistochemistry and Kaplan-Meier Analysis**.

To further validate the TCGA findings of prognostic value and cancer immunological role of SPTBN1 in KIRC, we collected KIRC tumor samples from 29 independent patients who were admitted at Sun Yat-sen University Cancer Center from Dec 2016 to Jun 2019 (SYSUCC KIRC cohort) for conducting validation immunohistochemistry (IHC) experiments. We collected tumor samples and survival data from patients who met the following eligibility criteria: (a) individuals had a pathological diagnosis of primary KIRC, and were excluded the presence of any other malignant disorders or any other metastatic tumor; (b) individuals had clear and complete baseline clinical data, including age, gender, tumor grade [International Society of Uro-Pathology (ISUP) grading system] and stage (TNM staging system), date of diagnosis and follow-up/death. This study was approved by the Ethical Committee of the Sun Yat-sen Cancer Center (Approval Number: B2022-472-01). Due to the retrospective study design, the Ethical Committee of the Sun Yat-sen Cancer Center approved a waiver of written informed consent to use the KIRC specimens. The specific IHC experimental methods of SPTBN1 immunostaining have been described in previous publications[27]. Briefly, all the 29 paraffin-embedded KIRC tissue samples were separated into two sections, one section of each tumor slice was subjected to immunostaining with 1:1000 diluted primary rabbit polyclonal anti-SPTBN1 (catalog number: 67978-1-Ig, ProteinTech®, Rosemont, IL, USA) overnight at 4 ℃, and the other section was immunostained with 1:100 primary rabbit anti-TNFSF9 diluent (catalog number: 66450-1-Ig, ProteinTech®, Rosemont, IL, USA) at 4 ℃ overnight. After washing and applying appropriate anti-rabbit secondary antibodies (catalog number: K3468, DAKO®, Carpinteria, CA) and diaminobenzidine (DAB) substrate mixture (catalog number: GK500710, GeneTech®, Shanghai, China) based on the manufacturer’s protocols, KIRC slides were visualized by using the Axioplan-2 imaging microscope (CarlZeiss®, Göttingen, Germany). Moreover, to determine the specificity of the primary SPTBN1/TNFSF9 antibodies, tumor slides were incubated in negative control rabbit IgG without the primary SPTBN1/TNFSF9 antibodies, and no non-specific immunostaining was found under this condition.

Then, semi-quantitative analysis of the IHC-protein expression (IHC-P) score was conducted by two independent researchers (WT and QS). In short, IHC-P score of each immunostained KIRC slide were assigned scores separately based on the stained area of IHC staining and the intensity of IHC staining. Quantitation of the IHC staining extent of SPTBN1 was made as follows: 1, < 33% of the tumor cells (sporadic); 2, 33 -66% of the tumor cells (focal); and 3, > 66% of the tumor cells (diffuse). The IHC staining intensity of SPTBN1 was scored as follows: 0, absent (no staining); 1, weak/moderate staining (light yellow or yellow brown); 2 strong staining (deep brown). Multiplication of the staining extent scores and staining intensity scores produces a SPTBN1 IHC-P score for each tumor slice, with a maximum score of 6 points (0–6 points), and the final IHC-P score of SPTBN1 was defined as follows: “-“, 0 point (negative); “+”, 1 point (weakly positive); “++”, 2–4 points (positive) and “+++”, 5–6 points (strong positive). Thus, based on the description of previous study[27], our 29 KIRC tissue samples from SYSUCC KIRC cohort were accordingly grouped to low SPTBN1-expression tumor tissue group (SPTBN1, “-“ and “+”, 0–1 point) and high SPTBN1-expression group (SPTBN1, “++” and “+++”, 2–6 points). On the other hand, the IHC staining intensity of TNFSF9 was scored from 0 to 3 points (0, no staining; 1, weak staining; 2, moderate staining and 3, strong staining), and the IHC staining extent of TNFSF9 was determined by the percentage of immunostained tumor cells ranging from 0 to 4 points (0, < 1%; 1, 1 − 10%; 2, 11 − 50%; 3, 51 − 80% and 4, > 80%) as described before[43]. The final IHC-P score of TNFSF9 ranging from 0 to 12 was derived from the score of the area of positive staining cancer cells × the score of IHC staining intensity, and defined as follows: “-“, 0 point (negative); “+”, 1–4 points (weakly positive); “++”, 5–8 points (positive) and “+++”, 9–12 points (strong positive).

To validate the prognostic role of SPTBN1 in KIRC based on our previous bioinformatic analysis, a Kaplan-Meier analysis of OS was performed for KIRC cases with low- and high-SPTBN1 expression patterns based on the IHC-P score from tumor tissue of SYSUCC KIRC cohort, and the last date of follow-up was 20 July 2022.

**Validation of the Correlation between SPTBN1 and TNFSF9 Expression, and the Prognostic Role of SPTBN1 in UVM Using Data from GEO database**.

To further confirm the TCGA findings of prognostic and immunological role of SPTBN1 in UVM, we used expression and survival data of GSE44295 dataset (Series Matrix File form), which was based on the GPL6883 platform of Illumina HumanRef-8 v3.0 expression bead-chip, consisting of 57 UVM patients with full-details of OS from Gene Expression Omnibus (GEO) database to achieve it[44]. As all validation analysis based on GSE44295 were dependent on obtaining explicit expression data of SPTBN1 and TNFSF9, it was necessary to transform the probes ID (ID_REF) in GSE44295 dataset into gene Name (gene_ symbol) based on the annotation file supplying by GPL6883 platform via a Perl script. All the expression profiling data in GES44295 were normalized using quantile normalization with background subtraction by its contributors before download. After transformation (ID_REF → gene_ symbol), the distributions of SPTBN1 and TNFSF9 raw expression abundances were demonstrated in a boxplot (revised_Supplementary File 1 A), and we observed that there were many unexplained extreme values in their distribution boxplots, indicating these GEO-downloaded raw expression data should be conducted further normalization before using in the following analysis. After performing log2(counts + 1) normalization via R package “limma“[40], the distributions of SPTBN1 and TNFSF9 expression abundances were shown in revised_Supplementary File 1B, and we could observe that the counts-values distributed almost evenly in their respective box plots without any extreme values, indicating the log2(counts + 1) normalization expression profiling of SPTBN1 and TNFSF9 in GSE44295 dataset could be used in the following validation analysis. Based on the median of SPTBN1 expression [log2(counts + 1) form], 57 UVM patients with known OS data in GSE44295 were divided into high- and low-SPTBN1 expression groups to further determine whether the expression of TNFSF9 and survival data between two groups had statistical differences.

### SPTBN1-related Gene Set Enrichment Analysis of Gene Ontology in KIRC and UVM

In order to analyze the biological functional pathway, gene set enrichment analysis (GSEA) was performed in the KIRC and UVM cases from TCGA database with low- and high-expression cohorts compared with the median level of SPTBN1 expression, respectively. The top 5 terms of gene ontology (GO) analysis in KIRC and UVM. Gene sets with NOM *p* value < 0.05 and FDR *q* value < 0.25 were regarded as significant enrichment[45].

### Construction of a SPTBN1-Associated Immunomodulator-Gene Prognostic signature in KIRC

Most researches with single genes have poor accuracy in clinical settings, so combining multiple genes in a pathway with a single gene to construct prognostic signatures are often used[46]. It could provide a more comprehensive and detailed picture of the clinical significance than only using a single gene. Thus, in current section, we aim to construct a prognostic signature by combining SPTBN1 with SPTBN1-associated immunomodulator-pathway genes for OS of KIRC under clinical settings. Firstly, by including these gene variables into a risk model via stepwise Cox regression analysis using R package “survival“[37], we can obtain an optimal prognostic gene signature. Then, we enter this gene signature with clinical settings (patients’ age, gender, tumor stage and grade) into a clinical risk model using univariate and multivariate Cox regression analysis to further determine whether this prognostic signature is an independent predictor for KIRC prognosis under clinical settings via R package “survival“[37]. Finally, to access the predictive value between this constructed gene signature and SPTBN1 alone, we perform a 3-years OS receiver operating characteristic (ROC) curve analysis to achieve them by using R packages “timeROC“[47].

### Evaluation of patients’ response to Immunotherapy in TCGA-KIRC with different expression levels of SPTBN1

Immunophenoscore (IPS) is an important indicator to evaluate patients’ response to immune check inhibitor (ICI) therapy. We therefore downloaded IPS data from The Cancer Immunome Atlas (TCIA) (https://tcia.at/home)[48] and used R package “limma“[40] to analyze and visualized differences in response to ICI therapy between low- and high-SPTBN1 expression groups in TCGA-KIRC cases.

### Potential sensitive drug prediction of SPTBN1 in TCGA-UVM

The half-maximal inhibitory concentration (IC50) is an important predictor for assessing the efficacy of an anti-cancer targeted drug or the response of a patient to anti-cancer targeted treatment. With the help of R package “pRRophetic“[49], differences in response (IC50) to anti-cancer targeted therapies between low- and high-SPTBN1 expression groups in TCGA-UVM cases were explored, and a difference of a drug with a cut-off of *p* value < 0.001 were selected to visualized.

### Statistical analysis

In TIMBER 2.0 platform, the analysis of differential mRNA expression of SPTBN1 in pan-cancer was conducted by using Wilcoxon test. The correlations between gene-gene expression or between gene-biomarkers of tumor immunity were assessed using Spearman’s analysis. In UALCAN platform, the analysis of differential expression of SPTBN1 at mRNA or protein levels in KIRC and UVM were performed by using Wilcoxon test. In R software (version 3.6.3) environment, Inter-group comparisons from TCGA database were performed for continuous variables using the Wilcoxon test for variables with a normal distribution and the Mann–Whitney *U-*test for those with a non-normal distribution. For survival analysis of TCGA, we used univariate Cox regression model and Kaplan-Meier curve with log-rank method to evaluate it. In following validation studies from our own cohorts and GEO dataset, all statistical analyses were conducted using IBM SPSS software (version 22.0) (IBM Inc., Armonk, NY, USA). Among them, analysis of SPTBN1 and TNFSF9 expression patterns in KIRC and UVM tumor samples were performed using Mann–Whitney *U-*test, and survival analysis of OS between high- and low-SPTBN1 expression groups was performed using Kaplan-Meier curve with log-rank method. Results with a *p* value < 0.05 were considered as statistical significance, if not specially noted.

## Results

### The expression of SPTBN1 was Dysregulated in Pan-cancer

**Fig. 1:**
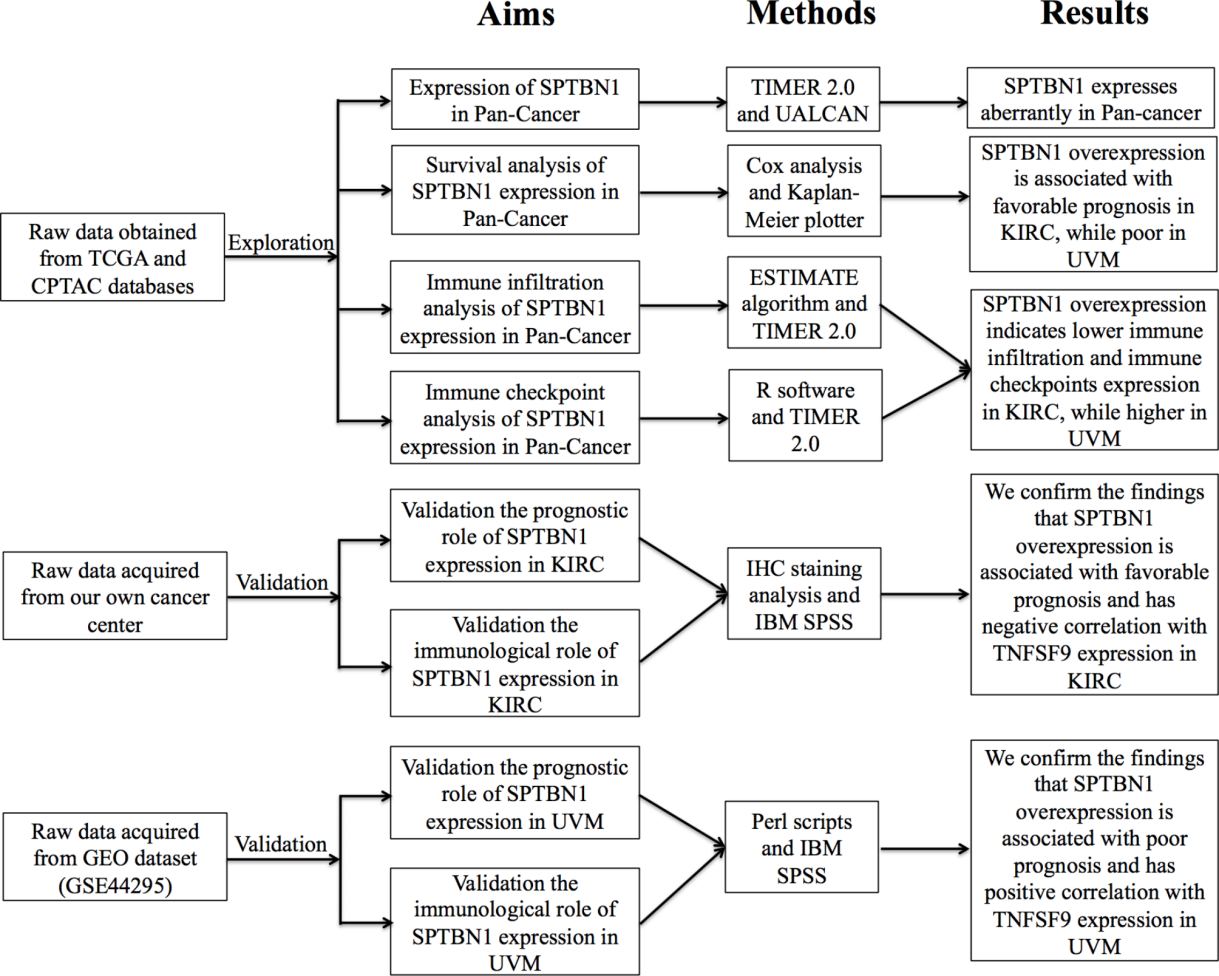
Flowchart for this study’s main contents. SPTBN1, non-erythrocytic spectrin beta 1; TCGA, the cancer genome atlas; CPTAC, clinical proteomic analysis consortium; GEO, gene expression omnibus; IHC, immunohistochemistry; KIRC, kidney renal clear cell carcinoma; UVM, uveal melanoma; TNFSF9, tumor necrosis factor superfamily member 9

**Fig. 2:**
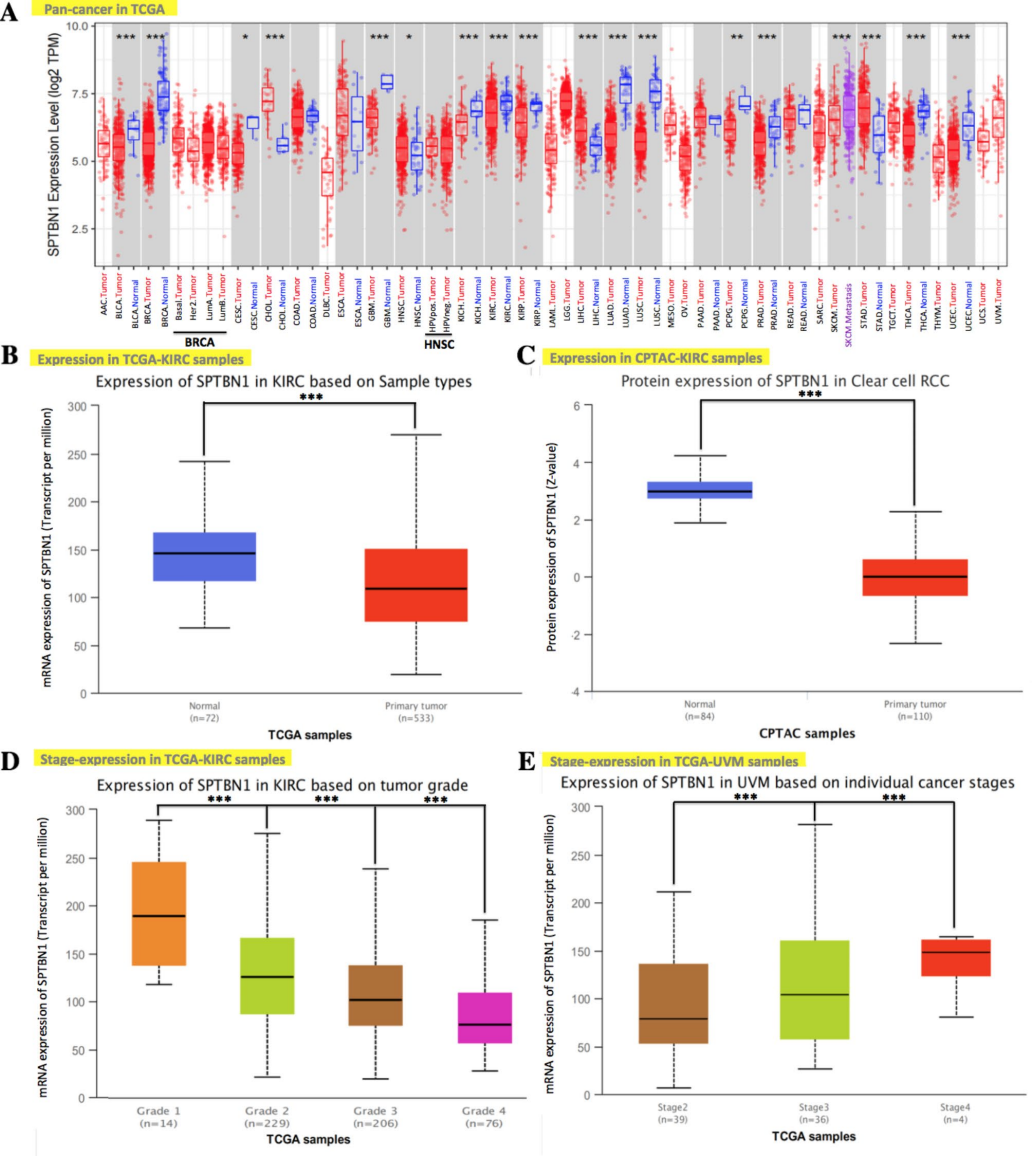
SPTBN1 aberrantly expressed in cancers. **(A)** Upregulated or downregulated expression levels (log2TPM unit) of SPTBN1 in different cancer types/subtypes from TCGA data in Pan-cancer in TIMER 2.0 platform. **(B)** Based on data from TCGA database, SPTBN1 mRNA expression levels (TPM unit) in tumor and corresponding normal tissues in KIRC in UALCAN platform. **(C)** Based on data from CPTAC dataset, the SPTBN1 protein expression status (Z-value unit) in primary tumor and normal tissues in KIRC in UALCAN platform. **(D, E)** SPTBN1 mRNA expression (TPM unit) in different tumor stages of KIRC and UVM from TCGA data in UALCAN platform. SPTBN1, non-erythrocytic spectrin beta 1; TCGA, the cancer genome atlas; CPTAC, clinical proteomic analysis consortium; KIRC, kidney renal clear cell carcinoma; UVM, uveal melanoma. UALCAN, University of ALabama at Birmingham CANcer. * *p* < 0.05; ** *p* < 0.01; *** *p* < 0.001

SPTBN1 has been described as a vital intracellular protein with multiple functions involving in integration of the cytoskeleton with ion channels, cell adhesion and ion transport[20]. In present study, we aimed to explore its function in cancers. The flowchart of the present research’s main contents was demonstrated in revised_Figure 1. Firstly, we used the TIMER2.0 online platform to explore the mRNA expression pattern of SPTBN1 across TCGA cancers. As shown in Fig. 2A, the mRNA expression levels of SPTBN1 in the cancer tissues of BLCA, BRCA, GBM, KICH, KIRC, LUAD, LUSC, PRAD, THCA, UCEC (all *p* < 0.001), PCPG (*p* < 0.01), and CESC (*p* < 0.05) were lower than the corresponding adjacent normal tissues, however, SPTBN1 mRNA expression in the tumor tissues of CHOL, LIHC, STAD (all *p* < 0.001), and HNSC (*p* < 0.05) were higher than their respective adjacent non-tumor tissues. Please note that due to the corresponding normal tissue samples in some tumor types, like ACC and UVM, were unavailable in TCGA database or CPTAC database, we were unable to perform differential expression analysis in these cancer types. Moreover, the downregulated expression of SPTBN1 in KIRC vs. corresponding adjacent normal tissues was further confirmed at mRNA and protein expression levels by using UALCAN online tool (Fig. 2B, C). Subsequently, SPTBN1 expression in different grades/stages of TCGA cancers were explored by using UALCAN platform. As shown in Fig. 2D, SPTBN1 were significantly downregulated in grade 4 (*p* < 0.001), grade 3 (*p* < 0.001) and grade 2 (*p* < 0.001) compared to grade 1 in KIRC patients. While, in UVM, the expression levels of SPTBN1 were obviously upregulated in stage 3 (*p* < 0.001) and stage 4 (*p* < 0.001) compared to stage 2 (Fig. 2E). These above results indicate that for different cancer types or tumor grades/stages, the expression of SPTBN1 in cancers is aberrant and quite different.

### Multifaceted prognostic value of SPTBN1 expression in cancers

**Fig. 3:**
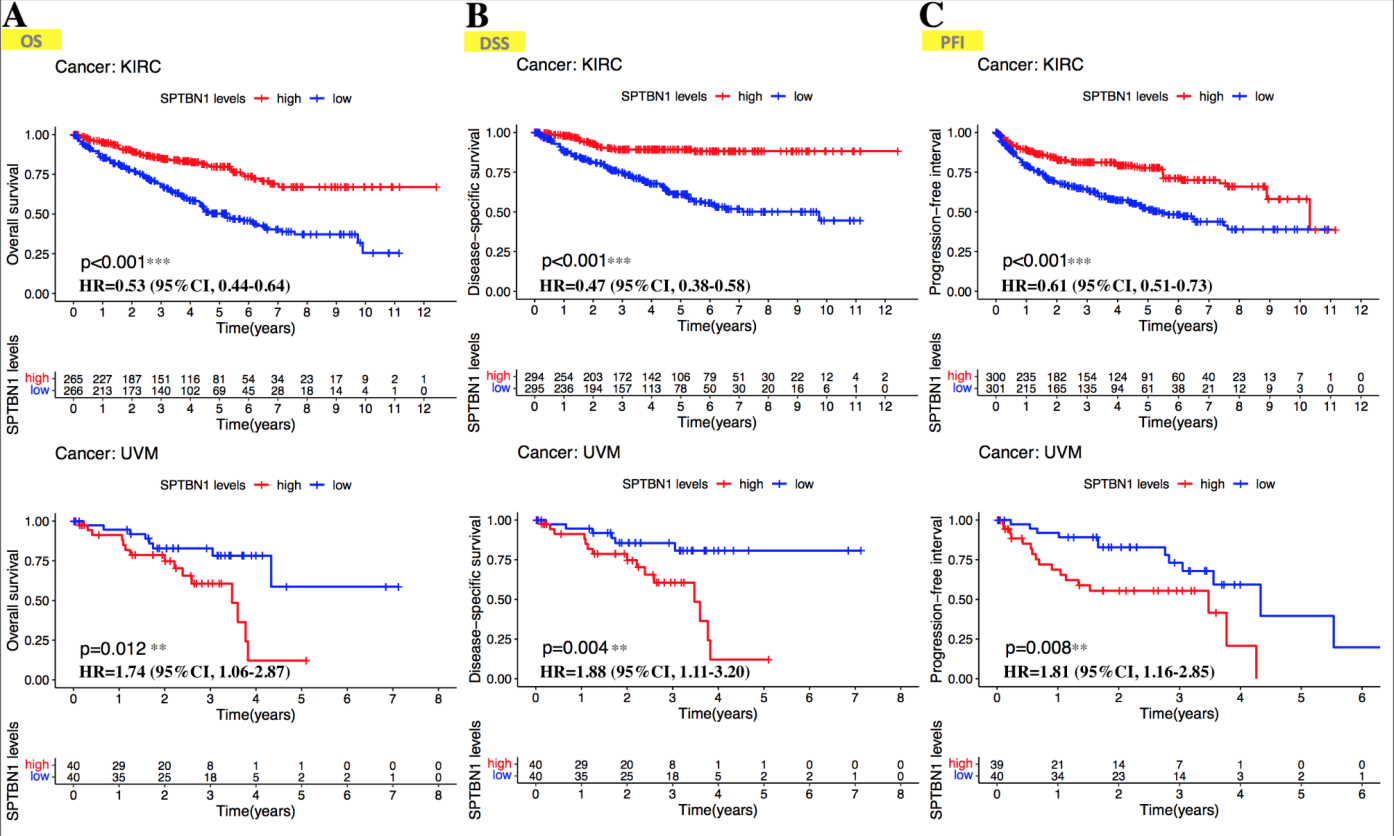
Kaplan-Meier survival curves comparing high and low expression of SPTBN1 in KIRC and UVM from TCGA data. Survival analysis of **(A)** OS, **(B)** DSS, and **(C)** PFI in KIRC (*upper*) and UVM (*lower*). The hazard ratios with 95% confidence intervals of SPTBN1 expression in Kaplan-Meier plots were calculated based on univariate Cox model. SPTBN1, non-erythrocytic spectrin beta 1; KIRC, kidney renal clear cell carcinoma; UVM, uveal melanoma; TCGA, the cancer genome atlas; OS, overall survival; DSS, disease-specific survival; PFI, progression-free interval; HR, hazard ratio; 95%CI, 95% confidence interval. ** *p* < 0.01; *** *p* < 0.001

We firstly analyzed the prognostic patterns of SPTBN1 in human cancers from TCGA database by using univariate Cox regression analysis. As demonstrated in Table 1, for OS, SPTBN1 had a protective effect on patients with KIRC (HR = 0.53, 95%CI, 0.44–0.64, *p* = 7.32E-12) and READ (HR = 0.49, 95%CI, 0.28–0.84, *p* = 0.01), and a detrimental effect on individuals with ACC (HR = 1.77, 95%CI, 1.04–2.99, *p* = 0.035), BLCA (HR = 1.26, 95%CI, 1.05–1.51, *p* = 0.014), CESC (HR = 1.67, 95%CI, 1.13–2.47, *p* = 0.011) and UVM (HR = 1.74, 95%CI, 1.06–2.87, *p* = 0.029). For DSS, SPTBN1 acted as a detrimental role in patients with ACC (HR = 1.94, 95%CI, 1.11–3.38, *p* = 0.02), CESC (HR = 1.73, 95%CI, 1.12–2.68, *p* = 0.014), LUSC (HR = 1.37, 95%CI, 1.08–1.74, *p* = 0.01) and UVM (HR = 1.88, 95%CI, 1.11–3.20, *p* = 0.019), and a protective role in patients with KIRC (HR = 0.47, 95%CI, 0.38–0.58, *p* = 3.83E-13) and THYM (HR = 0.09, 95%CI, 0.01–0.75, *p* = 0.026). For PFI, SPTBN1 played a protective role in cases with KIRC (HR = 0.61, 95%CI, 0.51–0.73, *p* = 8.19E-08), and a detrimental role in cases with ACC (HR = 2.74, 95%CI, 1.64–4.58, *p* = 0.0001), CESC (HR = 1.94, 95%CI, 1.33–2.85, *p* = 0.0006), PAAD (HR = 1.44, 95%CI, 1.06–1.97, *p* = 0.0209) and UVM (HR = 1.81, 95%CI, 1.16–2.85, *p* = 0.0095). Given the multifaceted prognostic role of SPTBN1 in various types of cancer, we subsequently focused on the role of SPTBN1 in KIRC and UVM. We divided the cancer patients into high-SPTBN1 expression and low-SPTBN1 expression groups based on the median expression level of SPTBN1 and explored the correlation of SPTBN1 expression with the prognosis of patients with KIRC and UVM from the TCGA database using Kaplan-Meier plotter. As shown in revised_Figure 3A, overexpression of SPTBN1 was associated with favorable prognosis of OS for KIRC (*p* < 0.001), while, bad prognosis of OS for UVM (*p* = 0.012). In addition, high expression level of SPTBN1 was linked to favorable prognosis of DSS (*p* < 0.001) and PFI (*p* < 0.001) for KIRC, however poor DSS (*p* = 0.004) and PFI (*p* = 0.008) for UVM (revised_Figure 3B, C). The above findings suggest that the level of SPTBN1 expression is a multifaceted factor affecting the survival of cancers, and in different types of human cancers, SPTBN1 may show different prognostic value in cancers.

**Table 1:** Univariate Cox analysis of OS, DSS and PFI in TCGA tumors between high and low expression of SPTBN1.

OS			DSS			PFI		
Cancer Type	HR (95%CI)	*P* Value	Cancer Type	HR (95%CI)	*P* Value	Cancer Type	HR (95%CI)	*P* Value
ACC	1.77 (1.04–2.99)	0.035*	ACC	1.94 (1.11–3.38)	0.020*	ACC	2.74 (1.64–4.58)	0.0001***
BLCA	1.26 (1.05–1.51)	0.014*	BLCA	1.24 (1.00-1.54)	0.050	BLCA	1.19 (0.99–1.42)	0.0583
BRCA	0.99 (0.77–1.27)	0.942	BRCA	1.17 (0.94–1.46)	0.149	BRCA	1.07 (0.90–1.27)	0.4232
CESC	1.67 (1.13–2.47)	0.011*	CESC	1.73 (1.12–2.68)	0.014*	CESC	1.94 (1.33–2.85)	0.0006***
CHOL	0.70 (0.43–1.17)	0.174	CHOL	0.72 (0.45–1.15)	0.171	CHOL	0.92 (0.60–1.41)	0.7038
COAD	0.94 (0.66–1.33)	0.710	COAD	0.98 (0.65–1.50)	0.937	COAD	1.06 (0.78–1.43)	0.7271
DLBC	1.29 (0.68–2.44)	0.430	DLBC	1.07 (0.43–2.68)	0.878	DLBC	1.28 (0.75–2.17)	0.3647
ESCA	1.02 (0.79–1.32)	0.891	ESCA	1.03 (0.76–1.39)	0.843	ESCA	1.02 (0.82–1.28)	0.8471
GBM	0.97 (0.75–1.25)	0.826	GBM	0.96 (0.74–1.25)	0.765	GBM	0.79 (0.59–1.05)	0.1033
HNSC	1.02 (0.84–1.24)	0.858	HNSC	0.95 (0.76–1.20)	0.675	HNSC	0.94 (0.78–1.13)	0.5024
KICH	0.70 (0.27–1.85)	0.475	KICH	0.77 (0.30–1.98)	0.586	KICH	0.73 (0.35–1.53)	0.4050
KIRC	0.53 (0.44–0.64)	7.32E-12***	KIRC	0.47 (0.38–0.58)	3.83E-13***	KIRC	0.61 (0.51–0.73)	8.19E-08***
KIRP	1.42 (1.00-2.02)	0.050	KIRP	1.49 (0.98–2.25)	0.059	KIRP	1.09 (0.82–1.43)	0.5625
LAML	0.92 (0.69–1.23)	0.585						
LGG	0.94 (0.64–1.39)	0.774	LGG	1.02 (0.67–1.54)	0.932	LGG	1.05 (0.77–1.42)	0.7553
LIHC	0.99 (0.78–1.27)	0.960	LIHC	1.00 (0.75–1.33)	0.978	LIHC	1.08 (0.89–1.31)	0.4190
LUAD	1.12 (0.91–1.39)	0.270	LUAD	1.08 (0.89–1.30)	0.448	LUAD	1.00 (0.86–1.16)	0.9985
LUSC	1.18 (0.92–1.51)	0.190	LUSC	1.37 (1.08–1.74)	0.010*	LUSC	1.20 (0.99–1.45)	0.0583
MESO	1.15 (0.82–1.61)	0.417	MESO	1.09 (0.72–1.66)	0.669	MESO	1.00 (0.70–1.44)	0.9902
OV	1.12 (0.92–1.36)	0.256	OV	1.10 (0.89–1.35)	0.366	OV	0.96 (0.80–1.15)	0.6538
PAAD	1.33 (0.96–1.85)	0.082	PAAD	1.37 (0.95–1.98)	0.093	PAAD	1.44 (1.06–1.97)	0.0209*
PCPG	1.46 (0.49–4.34)	0.500	PCPG	1.52 (0.44–5.28)	0.507	PCPG	1.09 (0.55–2.18)	0.8019
PRAD	0.91 (0.42–1.99)	0.821	PRAD	1.14 (0.32–4.05)	0.842	PRAD	0.90 (0.68–1.19)	0.4437
READ	0.49 (0.28–0.84)	0.010*	READ	0.47 (0.20–1.13)	0.093	READ	1.15 (0.67–1.96)	0.6086
SARC	0.96 (0.76–1.22)	0.734	SARC	0.91 (0.70–1.18)	0.472	SARC	0.88 (0.72–1.06)	0.1855
SKCM	0.94 (0.80–1.10)	0.434	SKCM	1.00 (0.84–1.19)	0.985	SKCM	0.94 (0.83–1.07)	0.3618
STAD	1.01 (0.82–1.23)	0.960	STAD	1.09 (0.85–1.41)	0.482	STAD	1.04 (0.84–1.28)	0.7226
TGCT	1.44 (0.24–8.59)	0.689	TGCT	1.19 (0.20–7.12)	0.850	TGCT	0.78 (0.48–1.27)	0.3204
THCA	2.45 (0.97–6.20)	0.059	THCA	2.49 (0.85–7.30)	0.096	THCA	0.84 (0.58–1.24)	0.3885
THYM	0.62 (0.24–1.59)	0.321	THYM	0.09 (0.01–0.75)	0.026*	THYM	0.90 (0.54–1.50)	0.6956
UCEC	0.91 (0.66–1.25)	0.556	UCEC	0.81 (0.55–1.18)	0.264	UCEC	0.83 (0.65–1.08)	0.1656
UCS	0.80 (0.46–1.39)	0.421	UCS	0.79 (0.44–1.41)	0.423	UCS	0.68 (0.41–1.12)	0.1315
UVM	1.74 (1.06–2.87)	0.029*	UVM	1.88 (1.11–3.20)	0.019*	UVM	1.81 (1.16–2.85)	0.0095**

OS, overall survival; DSS, disease-specific survival; PFI, progression-free interval; TCGA, the cancer genome atlas; SPTBN1, non-erythrocytic spectrin beta 1. * P < 0.05, ** P < 0.01, *** P < 0.001.

### Contradictory findings of KIRC and UVM in Correlations of SPTBN1 expression and Immune Infiltration

**Fig. 4:**
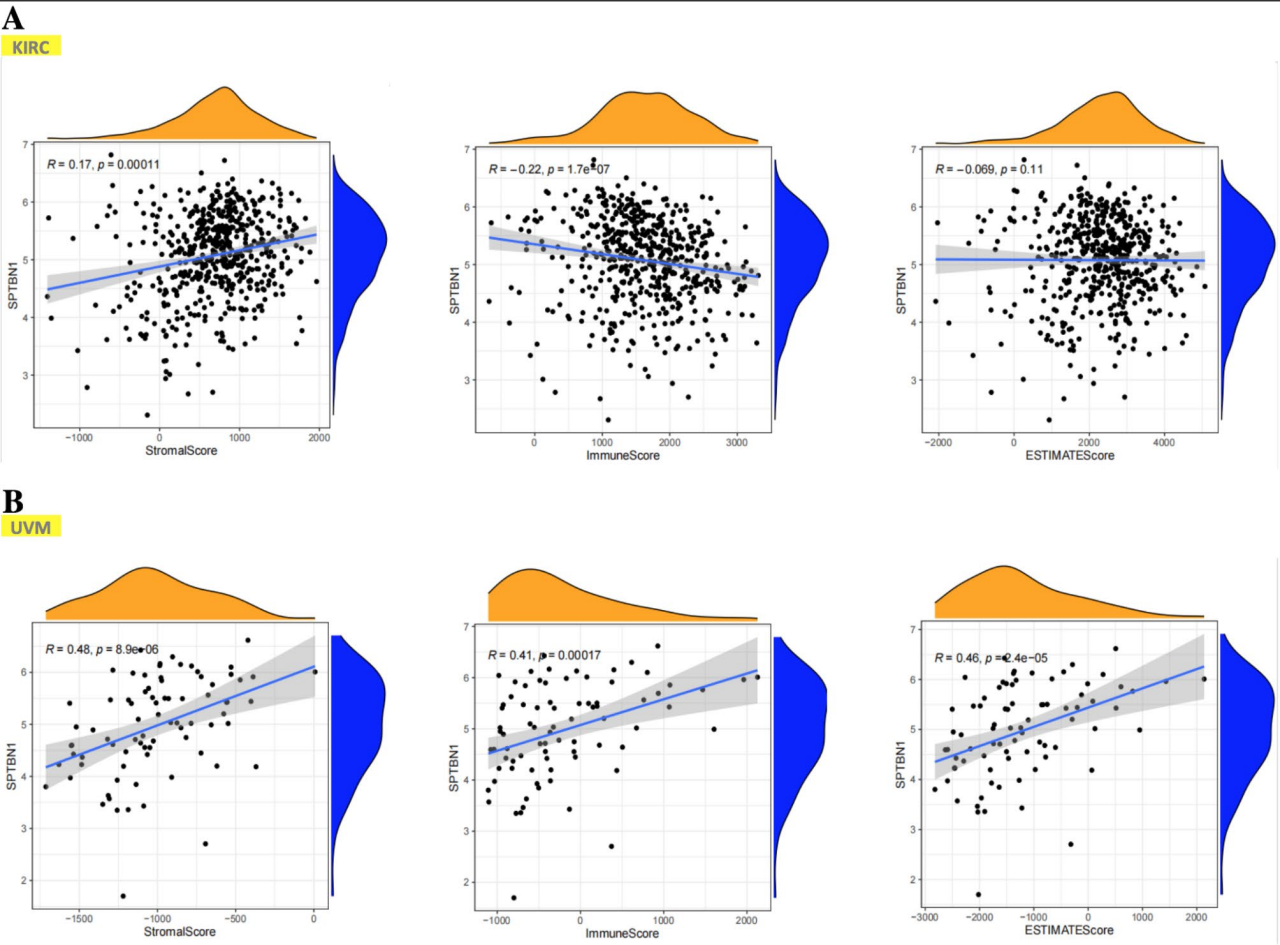
Analysis of the correlation of the estimated proportion of stromal and immune cells with SPTBN1 expression in tumor microenvironment in KIRC and UVM using ESTIMATE algorithm. **(A)** In KIRC, SPTBN1 expression was positively correlated with StromalScore (*left*), and negatively with ImmuneScore (*middle*) and not correlated with ESTIMATEScore (*right*). **(B)** In UVM, SPTBN1 expression was positively correlated with StromalScore (*left*), ImmuneScore (*middle*) and ESTIMATEScore (*right*). SPTBN1, non-erythrocytic spectrin beta 1; KIRC, kidney renal clear cell carcinoma; UVM, uveal melanoma. *p* value < 0.05 were considered to be statistically significant

**Fig. 5:**
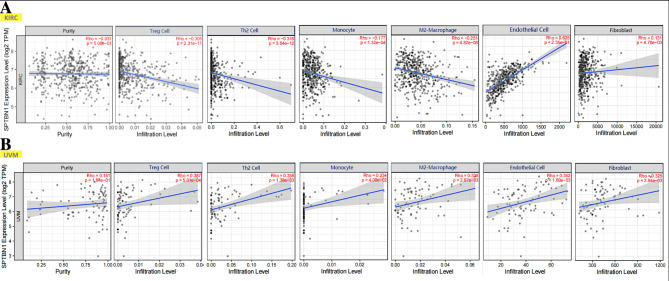
Analysis of the relationship between SPTBN1 expression and immune/stromal cell infiltration in KIRC and UVM using TIMER 2.0. **(A)** In KIRC, SPTBN1 expression was negatively correlated with immune cell infiltration, including Treg cell, Th2 cell, monocyte and M2-macrophage, and positively correlated with stromal cell infiltration, including endothelial cell and cancer-associated fibroblast. **(B)** In UVM, SPTBN1 expression was positively correlated with infiltration of immune cell and stromal cell, including Treg cell, Th2 cell, monocyte, M2-macrophage, endothelial cell and cancer-associated fibroblast. SPTBN1, non-erythrocytic spectrin beta 1; KIRC, kidney renal clear cell carcinoma; UVM, uveal melanoma. *p* value < 0.05 were considered to be statistically significant

It is widely noticed that the TME is a complicated mixture with various types of stromal cells and TIICs[30]. These cells act as a vital role in regulating the progression and development of cancers and affecting cancer patients’ survival. Recently, SPTBN1 is reported to be associated with many types of cancers for its essential roles in regulation of EMT and chronic inflammation in TME[20]. Thus, it is necessary to analyze the correlation between SPTBN1 expression and immune infiltration in tumors. Based on our above survival analysis results, we chose KIRC to represent tumors with favorable survival and UVM to represent tumors with poor survival when SPTBN1 reached an upregulated expression level. ESTIMATE algorithm was firstly used to analyze the correlation of SPTBN1 expression and estimated proportion of stromal/immune cells in TME in KIRC and UVM. For KIRC, the expression level of SPTBN1 had significant positive association with stromal cell proportion (R = 0.17, *p* = 0.00011) and significant negative association with immune cell proportion (R =-0.22, *p* = 1.7E-07) in TME (Fig. 4A). While, for UVM, high expression level of SPTBN1 had obvious positive correlations with the proportions of stromal cell (R = 0.48, *p* = 8.9E-06) and immune cell(R = 0.41, *p* = 0.00017) (Fig. 4B). Specifically, by using TIMER 2.0 online platform, we further found that the SPTBN1 expression had a significant positive association with the infiltration of stromal cells, including endothelial cells (R = 0.625, *p* = 2.35E-51) and cancer-associated fibroblast (R = 0.131, *p* = 4.78E-03), and a obvious negative association with the infiltration of TIICs, including Treg cells (R =-0.305, *p* = 2.31E-11), Th2 cells (R =-0.316, *p* = 3.54E-12), monocytes (R =-0.177, *p* = 1.32E-04) and M2-macrophage (R =-0.251, *p* = 4.82E-08) in KIRC after adjusting tumor purity (Fig. 5A). However, in UVM, overexpression of SPTBN1 positively correlated with the upregulated levels of infiltrating stromal cells, including endothelial cells (R = 0.352, *p* = 1.69E-03) and cancer-associated fibroblast (R = 0.325, *p* = 3.94E-03), and infiltrating immune cells, including Treg cells (R = 0.387, *P* = 5.01E-04), Th2 cells (R = 0.358, *p* = 1.38E-03), monocytes (R = 0.234, *p* = 4.08E-02) and M2-macrophage (R = 0.338, *p* = 2.67E-03) after adjusting tumor purity (Fig. 5B). These results in this section strongly suggest that SPTBN1 is tightly correlated with immune and stromal cell infiltration in TME and could affect individuals’ survival by interacting with immune infiltration. The relationships between SPTBN1 expression and immune infiltration in cancers could be positive or negative depending on the types of tumors.

### Relationships between SPTBN1 expression and Immune modulator markers in cancers

**Fig. 6:**
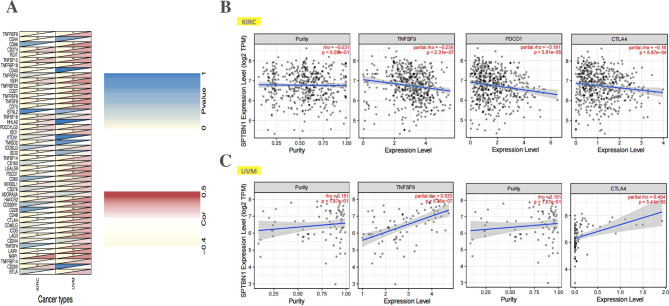
Analysis of the correlation between SPTBN1 expression and common immune modulator marker sets. **(A)** Correlation between SPTBN1 expression and 47 immune modulator marker sets across TCGA-KIRC and TCGA-UVM. Specifically, **(B)** In KIRC, SPTBN1 expression was negatively correlated with the expression of TNFSF9, PDCD1 and CTLA4. **(C)** In UVM, SPTBN1 expression was positively correlated with the expression of TNFSF9, PDCD1 and CTLA4. SPTBN1, non-erythrocytic spectrin beta 1; KIRC, kidney renal clear cell carcinoma; UVM, uveal melanoma; TNFSF9, tumor necrosis factor superfamily member 9; PDCD1, programmed cell death 1; CTLA4, cytotoxic T-lymphocyte-associated protein 4. * *p* < 0.05; ** *p* < 0.01; *** *p* < 0.001. *p* value < 0.05 were considered to be statistically significant

Immunosurveillance acts as an important role in affecting the prognosis and treatments of tumors. tumor cells could escape immunosurveillance via interacting with immune modulator markers, such as TNFSF9, CTLA4 and PDCD1, which are known as important molecules for cancerigenesis or cancer immunotherapy. The above results indicate that SPTBN1 could act as a multifaceted prognostic biomarker and a promising biomarker for immune infiltration in cancers, especially in KIRC and UVM. It has meaningful to explore the relationships between SPTBN1 expression and common immunomodulator genes in cancers. Thus, we explored relationships between SPTBN1 and 47 common types of immunomodulator genes in TCGA cancers (**revised_Suppmentary File 2**). Specifically, in TCGA-KIRC, SPTBN1 expression were negatively associated with the expression levels of 23 types of immunomodulator markers (all *p* < 0.05), and positively correlated with 8 types of immunomodulator genes expression (all *p* < 0.05). However, In TCGA-UVM, SPTBN1 expression were all positively correlated with the expression levels of 34 types of immunomodulator genes (all *p* < 0.05) (revised_Figure 6 A). by using TIMER 2.0 online tool and adjusting tumor purity, we found that SPTBN1 expression had negative correlations with the expression levels of TNFSF9 (R =-0.238, *p* = 2.31E-07), PDCD1 (R =-0.191, *p* = 3.81E-05) and CTLA4 (R =-0.16, *p* = 5.67E-04) in KIRC (revised_Figure 6B). On the contrary, in UVM, SPTBN1 expression had positive associations with the expression levels of TNFSF9 (R = 0.533, *p* = 5.95E-07), PDCD1 (R = 0.547, *p* = 2.65E-07) and CTLA4 (R = 0.454, *p* = 3.41E-05) (revised_Figure 6 C). All these results together strongly indicate that SPTBN1 expression is widely correlated with immunity in cancers.

**Validation of the Correlations Between SPTBN1 Expression and Immune Checkpoint Markers (TNFSF9) Expression in KIRC and UVM from Our Cancer Center and GEO Database**.

**Fig. 7:**
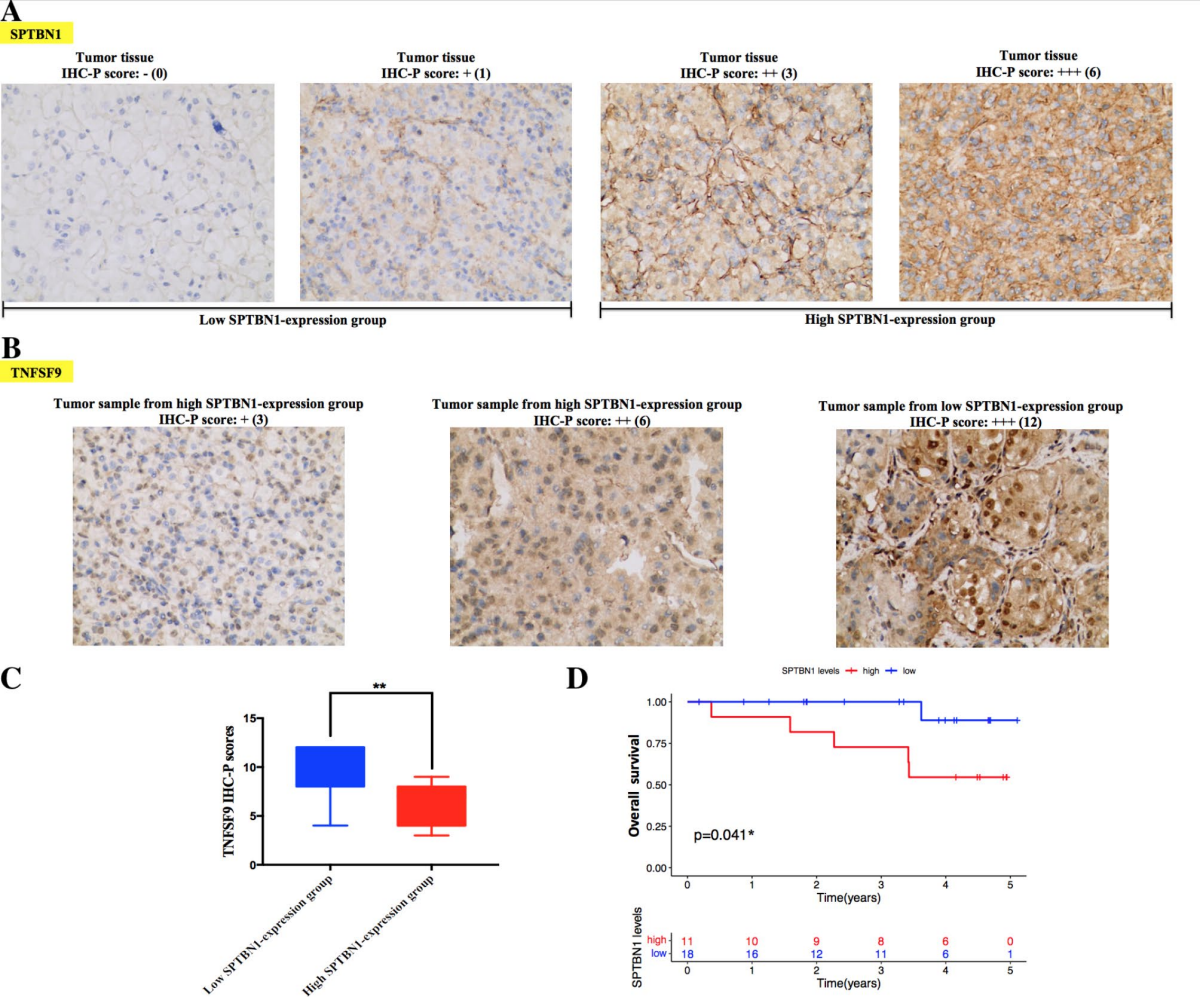
Validation analysis for determining the immunological and prognostic role of SPTBN1 in KIRC based on bioinformatic findings. **(A)** Representative images of different expression levels of SPTBN1 protein in KIRC tumor tissue samples via IHC staining. **(B)** Representative photomicrographs of IHC staining of TNFSF9 protein in KIRC tumor samples from high and low SPTBN1-expression groups. **(C)** The IHC-P scores of TNFSF9 in KIRC samples between high and low SPTBN1-expression groups were compared using Mann-Whitney *U*-test. **(D)** Overall survival analysis of clinical KIRC individuals between high and low SPTBN1-expression groups by using Kaplan-Meier curve with log-rank test. SPTBN1, non-erythrocytic spectrin beta 1; KIRC, kidney renal clear cell carcinoma; TNFSF9, tumor necrosis factor superfamily member 9; IHC, immunohistochemistry; IHC-P, immunohistochemistry protein expression. * *p* < 0.05; ** *p* < 0.01

As demonstrated in revised_Figure 7 A, IHC staining comparison of SPTBN1 expression status in tumor samples from clinical KIRC patients was performed. According to the staining results of IHC-P score of SPTBN1 from our own KIRC cases (SYSUCC KIRC cohort), we could group SYSUCC KIRC cohort (29 subjects) to high SPTBN1-expression KIRC group (18 subjects) and low SPTBN1-expression KIRC group (11 subjects). Then, to further validate the bioinformatic results that SPTBN1 expression was negatively associated with the expression of multiple immune modulator biomarkers in KIRC, we decide to detect expression of TNFSF9, an important immunotherapy checkpoint marker in KIRC, in tumor samples from high and low SPTBN1-expression KIRC groups using IHC staining (revised_Figure 7B). The IHC results demonstrated that the IHC-P score of TNFSF9 protein was significantly lower in high SPTBN1-expression group (18 cases) than those in low SPTBN1-expression group (11 cases) (Mann–Whitney *U* score = 39.5, *P* = 0.006). These IHC findings confirmed the above bioinformatic results that high expression of SPTBN1 had a significant negative correlation with the expression level of TNFSF9 in KIRC (revised_Figure 7 C and revised_Supplementary File 3).

**Fig. 8:**
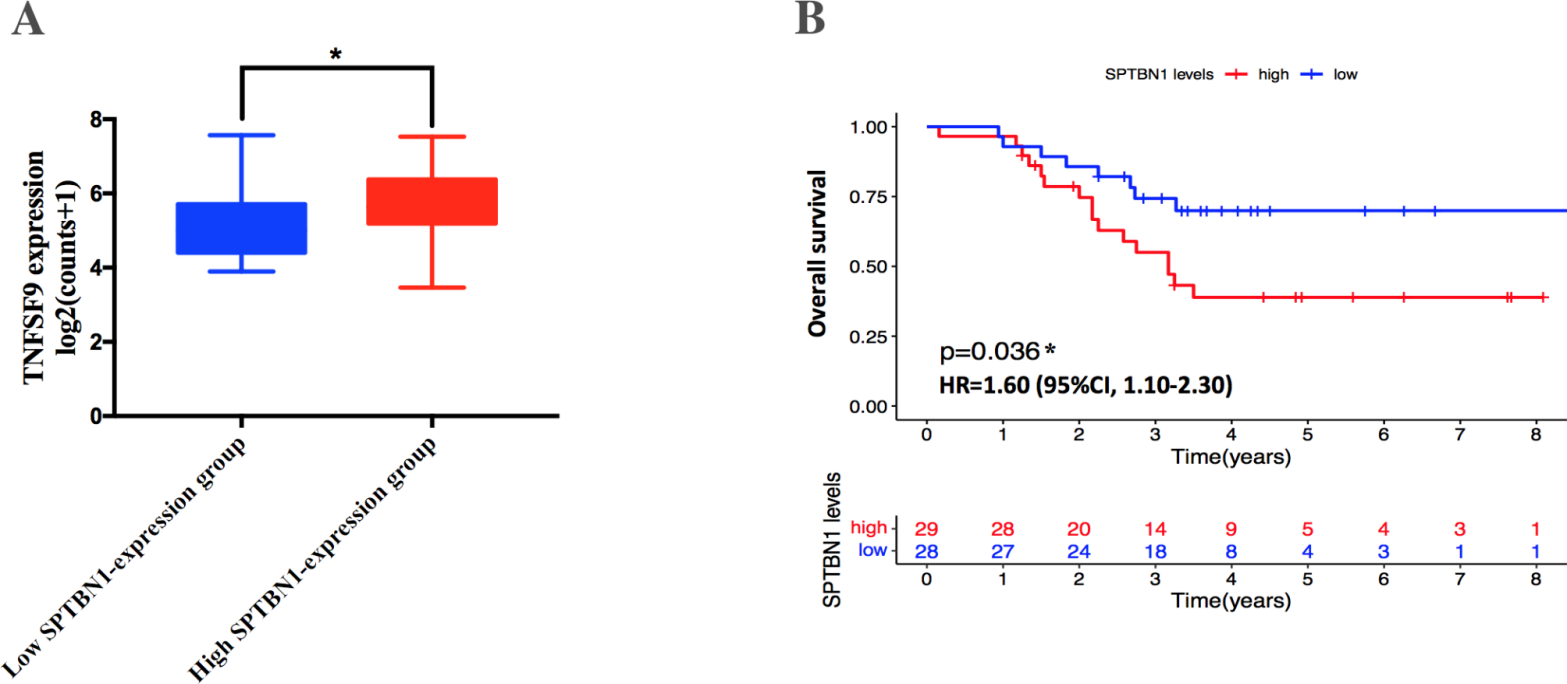
Validation study for confirming TCGA findings of SPTBN1 in UVM using data from GEO database. **(A)** TNFSF9 expression in UVM samples between high and low SPTBN1-expression groups from GSE44295 dataset were compared using Mann-Whitney *U*-test. **(B)** Overall survival analysis of UVM patients with high and low expression of SPTBN1 from GSE44295 dataset were determined by Kaplan-Meier curve with log-rank test. TCGA, the cancer genome atlas; SPTBN1, non-erythrocytic spectrin beta 1; UVM, uveal melanoma; GEO, gene expression omnibus; TNFSF9, tumor necrosis factor superfamily member 9. * *p* < 0.05

On the other hand, we also confirmed the reliability of previous TCGA results that SPTBN1 expression was positively associated with the expression of immune modulator markers related to immunotherapy in UVM. By using log2(counts + 1) normalization expression data of an independent UVM patient cohort (57 cases) from GEO database (GSE44295), we divided these patients into high SPTBN1-expression UVM group (29 cases, SPTBN1-mRNA expression level > 8.67) and low SPTBN1-expression UVM group (28 cases, SPTBN1-mRNA expression level < 8.67) according to the median expression level of SPTBN1-mRNA. The expression correlation between SPTBN1 and TNFSF9 in these two groups was shown in revised_Figure 8 A, and the results was in agreement with the findings in TCGA database. The expression level of TNFSF9 was also distinctly higher in tumor samples from high SPTBN1-expression UVM group compared to ones with low SPTBN1-expression level (Mann–Whitney *U* score = 245.0, *p* = 0.01). These GEO results validated the above TCGA findings that high expression of SPTBN1 had strong positive correlations with TNFSF9 expression in UVM (revised_Supplementary File 4).

**Validation of the Prognostic Roles of SPTBN1 in KIRC and UVM from Our Cancer Center and GEO Dataset**.

We have previously demonstrated that KIRC patients with high expression level of SPTBN1 exhibited favorable prognosis of OS in TCGA database. Following this, we conducted a retrospective survival analysis on our own KIRC subjects (SYSUCC KIRC cohort) to confirm whether high expression level of SPTBN1 could predict a favorable prognosis of individuals with KIRC. Survival study of the 29 KIRC cases was performed. As demonstrated in Fig. 7D, KIRC cases with high IHC-P score of SPTBN1 (high SPTBN1-expression KIRC group, 18 cases) had significant longer OS times compared to those with low IHC-P score of SPTBN1 (low SPTBN1-expression KIRC group, 11 cases) (Log Rank = 4.184, *p* = 0.041) (revised_Supplementary File 3), which was in agreement with the previous bioinformatic findings in patients with KIRC from TCGA database.

We also performed a clinical survival analysis on UVM patients from GSE44295 to validate TCGA findings that high expression level of SPTBN1 could predict a poor prognosis of patients with UVM. As shown in revised_Figure 8B, we found UVM cases with high levels of mRNA expression of SPTBN1 in tumor samples (high SPTBN1-expression UVM group, 29 subjects) had obvious shorter OS times than that with low expression levels of SPTBN1 (low SPTBN1-expression UVM group, 28 subjects) (Log Rank = 4.357, *p* = 0.036) (revised_Supplementary File 4), which was consistent with our previous TCGA findings in UVM patients.

### GO Enrichment Analysis of SPTBN1 in KIRC and UVM

**Fig. 9:**
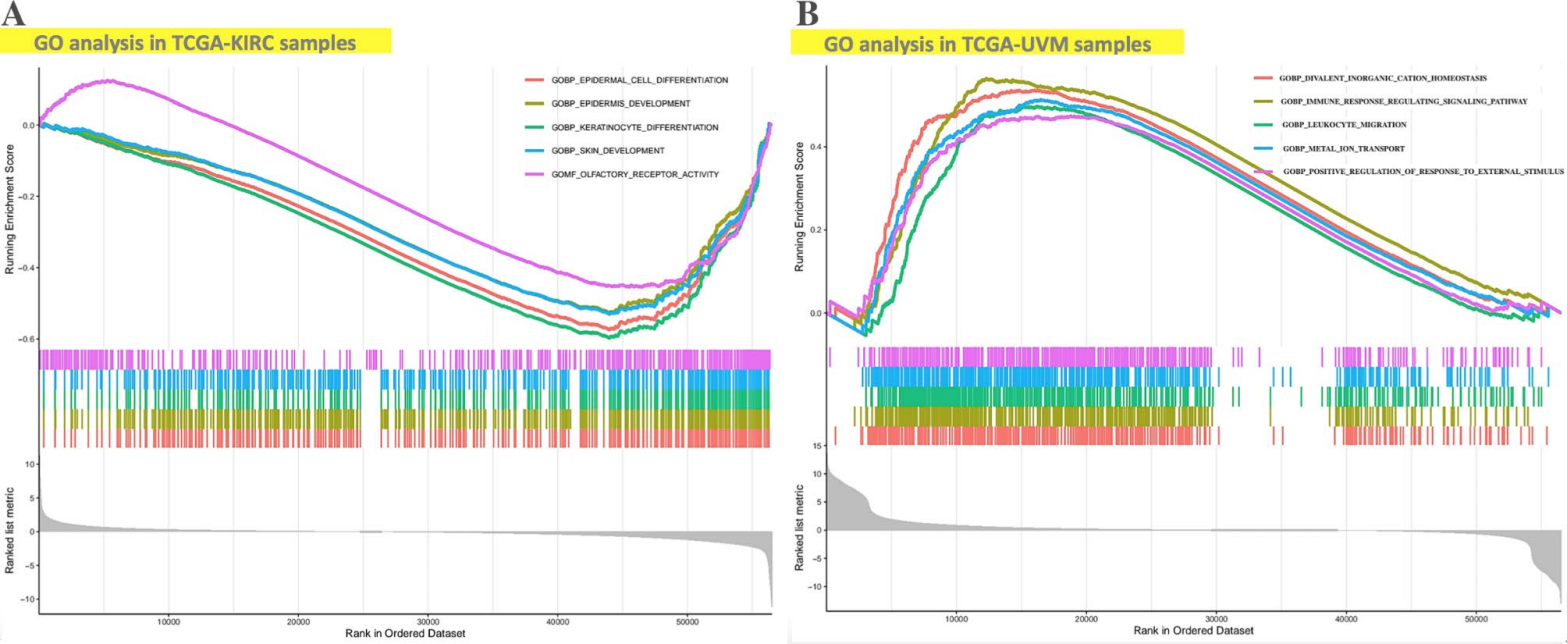
GO analysis of SPTBN1 expression in TCGA-KIRC and TCGA-UVM samples based on GSEA. **(A)** The enriched gene sets in GO collection via low expression of SPTBN1 in KIRC samples. **(B)** The enriched gene sets in GO collection by high expression of SPTBN1 in UVM samples. Each line showing one specific gene set with unique color. Downregulated gene sets gathered in the right of x-axis of the plot; while, upregulated gene sets located in the left of x-axis of the plot. Only top 5 gene sets with NOM *p* < 0.05 and FDR *q* < 0.25 were shown in the plot. GO, gene ontology; SPTBN1, non-erythrocytic spectrin beta 1; TCGA, the cancer genome atlas; KIRC, kidney renal clear cell carcinoma; UVM, uveal melanoma; GSEA, gene set enrichment analysis

To explore the potential different functional roles that SPTBN1 took part in the carcinogenesis of KIRC and UVM, we used GSEA to determine it. As demonstrated in revised_Figure 9 A, GO enrichment term revealed that low expression of SPTBN1 in TCGA-KIRC samples could promote KIRC progression mostly via affecting the process of epidermoid cell differentiation; while, in TCGA-UVM samples, high expression level of SPTBN1 could involve in the carcinogenesis of UVM mainly by dysregulating immunity-related activities, such as immune response signaling pathway and leukocyte migration, and electrolyte homeostasis, like metal-ion transportation and divalent ions homeostasis (revised_Figure 9B). These elements based on GO functional analysis probably provide some information to explain our above clinical findings that downregulation of SPTBN1 was associated with poor prognosis in KIRC patients, and upregulation of SPTBN1 was related to poor prognosis in UVM patients.

### The Prognostic implication of SPTBN1-Associated Immunomodulators in KIRC

**Fig. 10:**
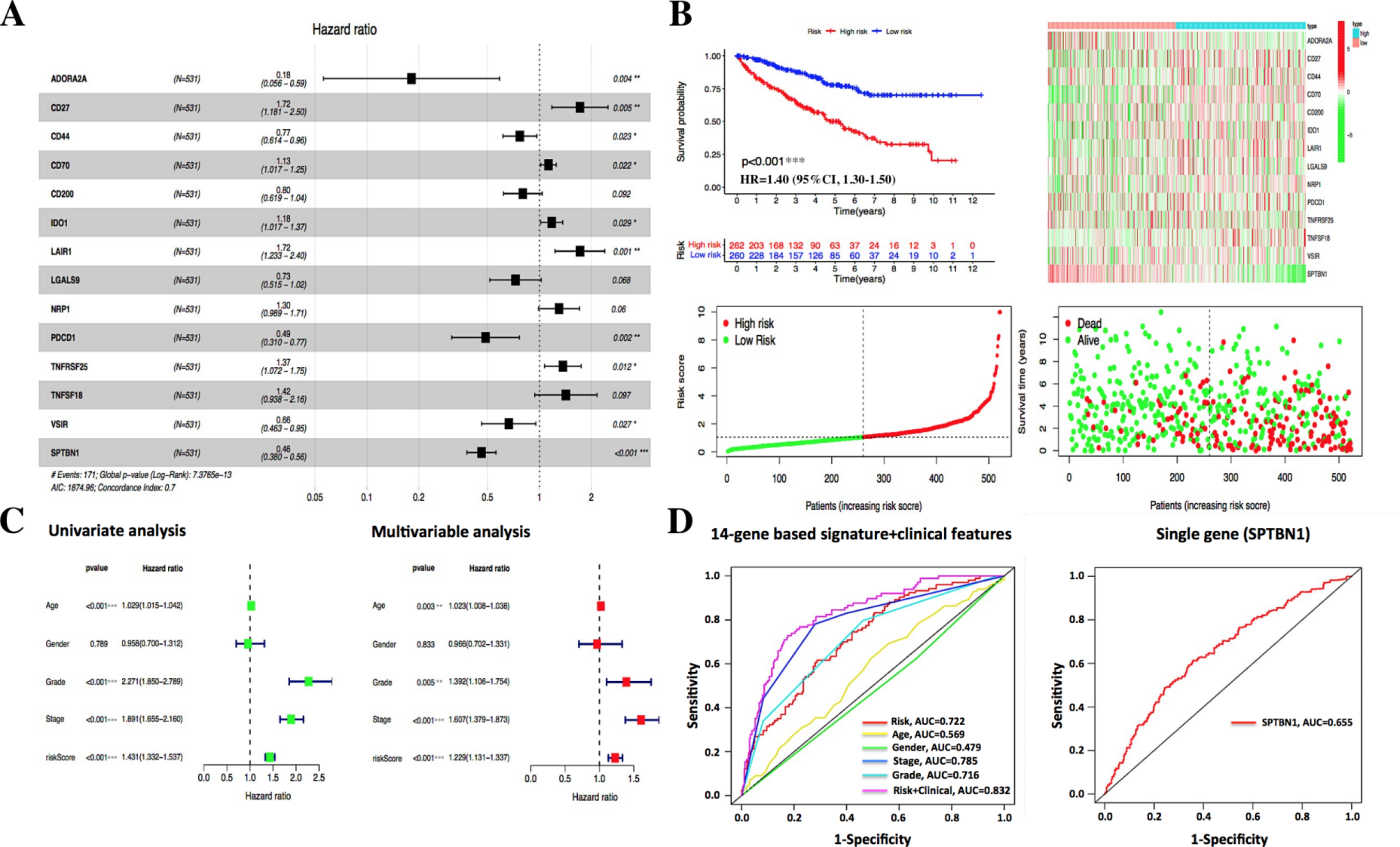
Development and evaluation of the prognostic gene signature based on SPTBN1-associated immunomodulators and SPTBN1. **(A)** The HR of genes integrated into the optimal prognostic 14-gene signature based on the stepwise Cox regression method are displayed in the forest plot for KIRC. **(B)** Prognostic evaluation of the 14-gene signature model in KIRC based on the risk score, including Kaplan-Meier curve, distribution of risk score along with survival status, and gene expression heatmap. **(C)** Univariate and multivariate Cox regression analyses of the risk score along with clinical features (age, gender, tumor grade and stage) in KIRC. **(D)** Time-dependent ROC curves at 3-years for evaluating risk scores based on 14-gene signature and single gene SPTBN1 in KIRC. SPTBN1, non-erythrocytic spectrin beta 1; HR, hazard ratio; KIRC, kidney renal clear cell carcinoma; ROC, receiver operating characteristic. * *p* < 0.05; ** *p* < 0.01; *** *p* < 0.001

To explore the prognostic value of SPTBN1-associated immunomodulators in KIRC, we entered those 31 immunodulator gene (revised_Figure 6 A) with SPTBN1 into a stepwise multivariate Cox regression model and generated an optimal prognostic 14-gene signature (ADORA2A-CD27-CD44-CD70-CD200-IDO1-LAIR1-LGALS9-NRP1-PDCD1-TNFRSF25-TNFSF18-VSIR-SPTBN1) for KIRC. The correlations between these 14 genes and OS of KIRC were presented in revised_Figure 10 A. The risk score was calculated by adding up the expression value and coefficient of each gene and could divide KIRC patients into high- and low-risk groups (revised_Supplementary File 5). The Kaplan-Meier survival curve elucidated those cases with low-risk had significant longer survival time than those with high-risk (HR = 1.40, 95%CI, 1.30–1.50, *p* < 0.001); meanwhile, the distribution plots of risk score, survival status and signature gene expression heatmap also demonstrated a good predictive value of this prognostic signature (revised_Figure 10B). As shown in revised_Figure 10 C, the risk score based on this prognostic signature was obviously associated with survival in KIRC in the univariate Cox model (HR = 1.431, 95%CI, 1.33–1.54, *p* < 0.001). More importantly, multivariate Cox model showed that the risk score based on 14-gene signature was an independent predictor of prognosis in KIRC after adjusting for clinical settings, including age, gender, tumor grade and stage (HR = 1.229, 95%CI, 1.131–1.337, *p* < 0.001). The area under the 3-years ROC curve (AUC) value of the risk score based on 14-gene signature was 0.722, and when combining above clinical features, the AUC value could reach 0.832; however, when using risk score based on SPTBN1 alone via stepwise Cox regression analysis (revised_Supplementary File 6), the AUC value was only 0.655, indicating that analyzing a single gene has limitations on prognostic prediction compared to a pathway-associated gene signature in clinical settings (revised_Figure 10D).

### Analysis of SPTBN1 expression for Predicting Immunotherapy response in patients with KIRC

**Fig. 11:**
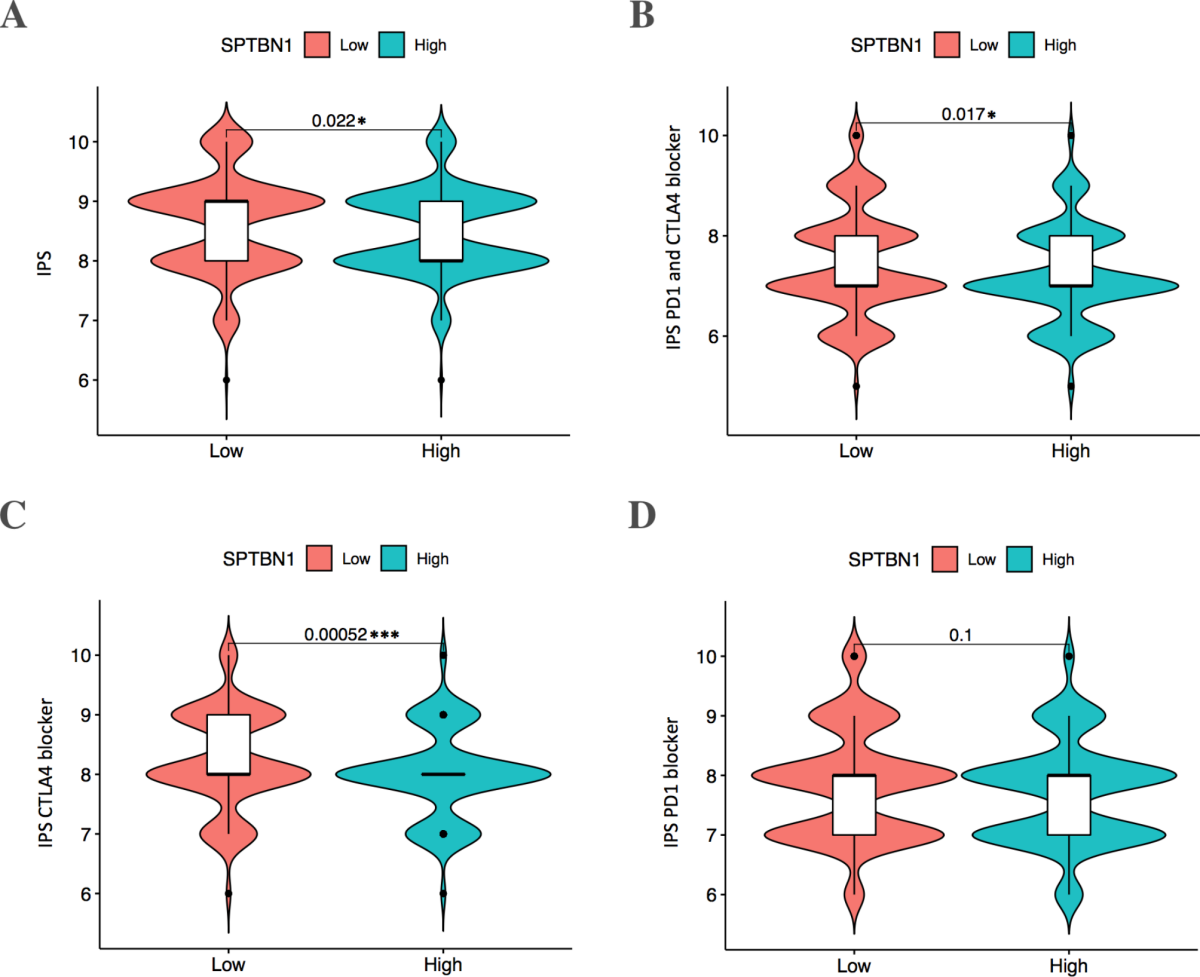
Violin plots illustrated the comparisons of the **(A)** IPS, **(B)** IPS-PD1 plus CTLA4 blockers, **(C)** IPS-CTLA4 block, and **(D)** IPS-PD1 blocker between low- and high-SPTBN1 expression TCGA-KIRC patient groups. IPS, immunophenoscore; TCGA, the cancer genome atlas; KIRC, kidney renal clear cell carcinoma; SPTBN1, non-erythrocytic spectrin beta 1; PD1, programmed cell death 1; CTLA4, cytotoxic T-lymphocyte-associated protein 4. * *p* < 0.05; *** *p* < 0.001

Additionally, the correlation between SPTBN1 expression and immunotherapy response (ICI therapy) in KIRC patients was analyzed via TCIA and TCGA databases using IPS evaluation method (revised_Supplementary File 7 and File 8). Revised_Figure 11 A-C demonstrated that IPS, IPS-CTLA4 plus PD1 blockers, and IPS-CTLA4 blocker were significantly higher in low-SPTBN1 expression KIRC group compared with those with high-SPTBN1 expression (all *p* < 0.05); while, IPS-PD1 blocker showed no significant difference between two groups (*p* = 0.1, Revised_Figure 11D). These results indicated that SPTBN1 was potentially involved in the resistance of immunotherapy of certain ICI therapy combinations, including CTLA-4 blocker and CTLA-4 plus PD1 blockers, in KIRC. We speculate that KIRC patients with high expression level of SPTBN1 might not gain enough benefit from ICI therapy.

### Analysis of SPTBN1 expression for Predicting Response of Anti-Cancer targeted treatment in patients with UVM

**Fig. 12:**
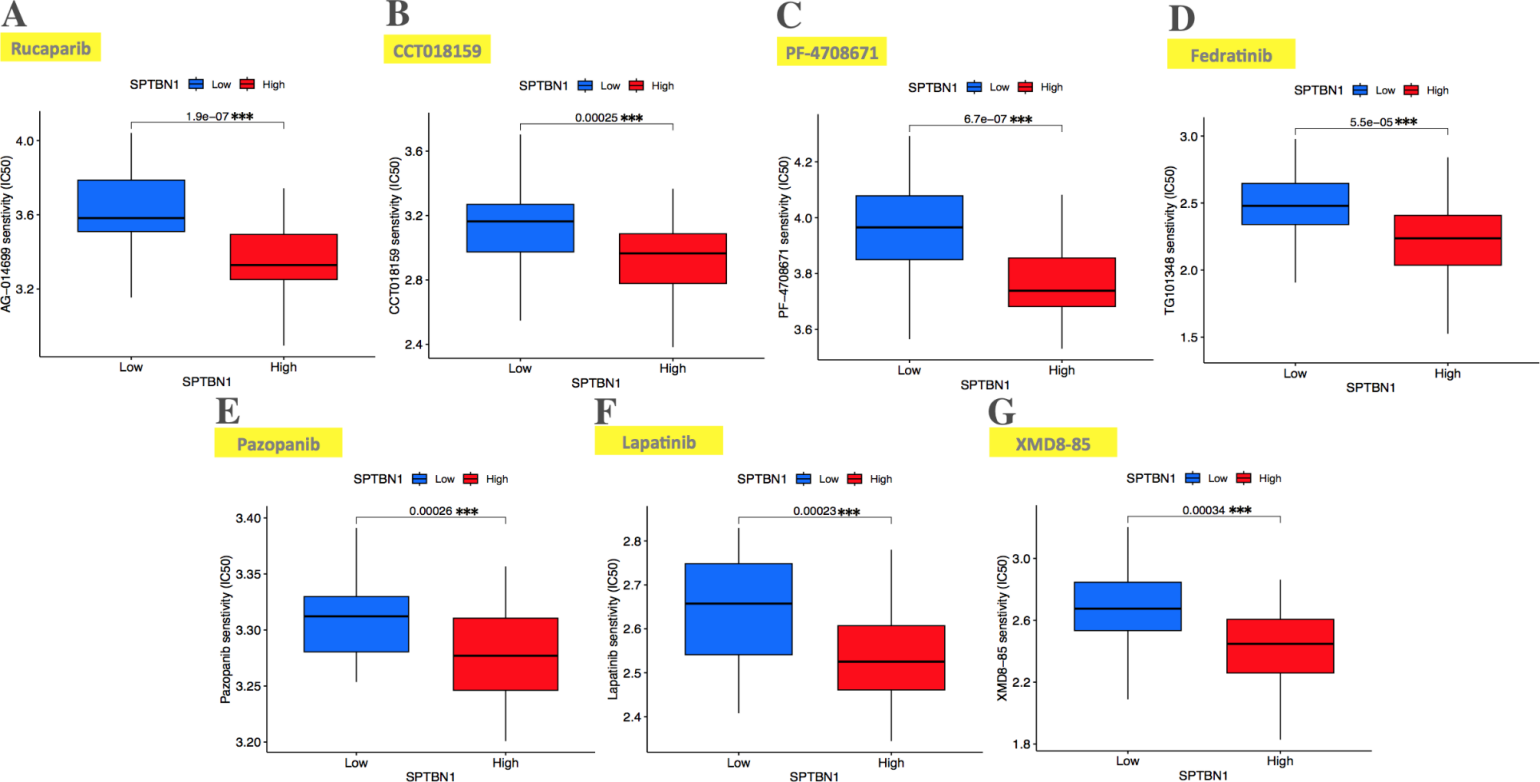
Box plots demonstrated the comparisons of IC50 scores of anti-cancer targeted drugs in low- and high-SPTBN1 expression TCGA-UVM patient groups via pRRophphetic R package. **(A)** Rucaparib. **(B)** CCT018159. **(C)** PF-4,708,671. **(D)** Fedratinib. **(E)** Pazopanib. **(F)** Lapatinib. **(G)** XMD8-85. IC50, half-maximal inhibitory concentration; TCGA, the cancer genome atlas; UVM, uveal melanoma; SPTBN1, non-erythrocytic spectrin beta 1. *** *p* < 0.001

On the other hand, the correlation between SPTBN1 expression and anti-cancer targeted treatment response in TCGA-UVM patients was explored using IC50 score. Revised_Figure 12 A-G showed that the IC50 scores of Rucaparib, CCT018159, PF-4,708,671, Fedratinib, Pazopanib, Lapatinib, and XMD8-85 were lower in the SPTBN1 high-expression UVM group compared with those in low-expression group (all *p* < 0.001), reflecting that UVM patients with high expression of SPTBN1 may exhibit a favorable response to anti-cancer targeted therapy.

## Discussion

Recently, more and more studies indicated that dysregulation of SPTBN1 had close links with many kinds of human disease, including congenital organ anomalies[16] and cancers[20]. However, the exact function of SPTBN1 in cancer progression and therapy has not been well-studied and still need further illumination. Through searching literatures in PubMed database, we could not find any literature associated with pan-cancer analysis of SPTBN1. Thus, we prepared this study to explore the prognostic and immunological role of SPTBN1 in pan-cancer, especially in KIRC and UVM.

Previous publications suggested that downregulation of SPTBN1 expression was frequently found in many kinds of cancers[20]. In present study, by using TIMER 2.0 web-based platform to explore the expression patterns of SPTBN1 across TCGA cancers at mRNA level, we revealed the consistent findings that SPTBN1, compared to expression level in nontumor adjacent tissues, was frequently down-expressed in tumor tissues in most types of cancers, including BRCA, BLCA, CESC, GBM, KICH, KIRC, KIRP, LUAD, LUSC, PCPG, PRAD, THCA and UCEC. In addition, by integrating independent databases of TCGA and CPTAC, we further validated that the expression of SPTBN1 was significantly downregulated in tumor tissues compared with normal tissues in KIRC patients at both mRNA and protein expression levels. Moreover, based on TIMER 2.0 online platform, we also found that with the tumor progression, the expression of SPTBN1 was gradually decreased in KIRC cases; meantime, in UVM patients, the expression level of SPTBN1 was gradually increased (Fig. 1), implying that SPTBN1 expression is closely related to tumor types and stages/grades, and it should be noted that the role of SPTBN1 in cancer development may vary depends on different contexts.

On the other hand, we applied a Cox regression analysis to evaluate the prognostic value (OS, DSS and PFI) of SPTBN1 expression across TCGA cancers. The analysis revealed that upregulated SPTBN1 expression could be used as a poor prognosis factor for OS, DSS and PFI in ACC, CESC and UVM. In contrast, in KIRC, a low expression level of SPTBN1 was indicated to be associated with a poor prognosis factor for OS, DSS and PFI (Table 1). In addition, we used another method, Kaplan-Meier analysis, to perform a survival analysis to further evaluate the prognostic value of SPTBN1 in KIRC and UVM, and the results were in agreement with the findings of Cox regression analysis that decreased expression of SPTBN1 was closely associated with poor prognosis of OS, DSS and PFI in KIRC, and favorable prognosis of OS, DSS and PFI in UVM (Fig. 3). Taken together, these results strongly demonstrate that in different kinds of cancers, such as KIRC and UVM, SPTBN1 can serve as a multifaceted indicator with different prognostic value. To the best of our knowledge, there is no study has found the direct links between SPTBN1 expression and prognosis of KIRC and UVM. By using tumor samples obtained from our cancer center and expression data obtained from independent dataset GSE44295, our following validation studies proved for the first time that low expression of SPTBN1 was directly linked to an unfavorable clinical prognosis in KIRC patients, and a favorable clinical prognosis in UVM cases (Figs. 7 and 8). Although present study may offer a novel view associated with cancer patients’ survival, especially in patients with KIRC and UVM; however, considering the contradictory prognostic roles of SPTBN1 in different cancer types, possible mechanisms involved in SPTBN1 regulated tumor progression are still needed discussing. As previous publications shown, SPTBN1 could play multifaceted roles in different kinds or stages of cancers via functioning in DNA repair to inhibit tumor progression[21] and in EMT to promote tumor progression[25]. We speculated that in early grades of primary tumor with low aggressiveness manner, like KIRC, high expression level of SPTBN1 could exert its anti-cancer effect through DNA repair in damaged tissue; while, with the primary tumor progression, the expression level of SPTBN1 was reduced accordingly and the anti-cancer effect was gradually disappeared. On the other hand, in cancer with high aggressiveness manner that could metastasize extensively at early stage, like UVM, high expression level of SPTBN1 could exert its pro-cancer effect via EMT to promote tumor metastasis since early stage. With cancer metastasis progressing, the expression level of SPTBN1 was further increased and the pro-cancer effect was gradually amplified. Moreover, GO functional analysis based on GSEA demonstrated that downregulation of SPTBN1 could exert its pro-cancer effects via affecting normal epidermoid cell differentiation in KIRC; while, upregulation of SPTBN1 could also exert its pro-cancer effects by dysregulating normal immunity-related activities, and electrolyte homeostasis in UVM (revised_Figure 9). These speculations and GO findings may help explain contradictory roles of SPTBN1 in different kinds of cancers. However, whether SPTBN1 could exert different roles and mechanisms in cancer development for different cancer types or stages still need additional experiments to prove it.

TME is a complicated milieu with a full of nontumor cells around cancer cells. Among these noncancerous cells, infiltrated immune cells, such as Treg cell, Th2 cell, monocyte and M2-macrophage, play an important role in the protumor immune response, including immune escape and cancer metastasis[30]. So far, it is unclear whether SPTBN1 expression has close relationship with tumor infiltration of immune cells. As demonstrated in Figs. 4 and 5, we did find significant associations between SPTBN1 expression in tumor immune cells infiltration in KIRC and UVM. Intriguingly, SPTBN1 overexpression in KIRC was negatively correlated with the invasion of pro-tumor immune cells; however, in UVM, the correlation was positive, implying SPTBN1 expression in KIRC and UVM may interact with immune infiltration in different manners and this finding could also help explain contradictory outcomes in cancer patients’ survival. The different interaction manners between SPTBN1 expression and infiltration of pro-tumor immune cells in different kinds of cancers could provide novel insights into understanding of the paradoxical role of SPTBN1 in affecting the occurrence, development and metastasis of tumor.

In contrast, cancer immunotherapy can recover the normal body’s function of antitumor immune response via regulating the expression of checkpoint genes, such as TNFSF9, PDCD1 and CTLA4. Here, we collected 47 common immune modulator genes, and analyzed the correlations between SPTBN1 expression and expression of these immune modulator genes across TCGA cancers (revised_Supplementary File 2 and revised_Figure 6 A). We found that in KIRC, SPTBN1 expression was negatively associated with the expression of more than 20 types of immune modulator genes; while, in UVM, SPTBN1 expression was positively associated with the expression of more than 30 types of immune modulator genes. Among these immune modulator genes, the expression levels of TNFSF9, PDCD1 and CTLA4 were all negatively correlated with SPTBN1 expression in KIRC, and positively correlated with SPTBN1 expression in UVM (revised_Figure 6B, C). These findings could also help explain the contradictory prognosis value of SPTBN1 in KIRC and UVM. Importantly, these findings shed light on understanding the potential role of SPTBN1 in cancer immunity. Moreover, further analysis of SPTBN1 for predicting ICI therapy response in patients with KIRC revealed that SPTBN1 was potentially involved in the resistance of certain ICI therapy, such as CTLA-4 blocker and CTLA-4 plus PD1 blockers in KIRC(revised_Figure 11); meanwhile, further study of SPTBN1 for predicting anti-cancer targeted therapy response in individuals with UVM demonstrated that SPTBN1 might also have a potential to involve in the enhance of anti-cancer targeted treatment in UVM (revised_Figure 12). These results revealed that SPTBN1 has a potential to serve as a biomarker not only for immunotherapy, but also for anti-cancer targeted treatment. However, additional prospective clinical studies based on real-world are needed in future for confirming it. For further proving these bioinformatic findings based on TCGA, we then performed a series of validation experiments (Figs. 7 and 8) and the results were in agreement with previous TCGA findings that SPTBN1 expression had negative and positive relationships with TNFSF9 expression in KIRC and UVM, respectively, further suggesting its potential to be a novel biomarker for anti-cancer therapies and prognosis in KIRC and UVM.

Yet it’s worth noting that the tumor suppressing and survival predicting roles of SPTBN1 in many types of human solid adenocarcinoma. For examples, by using weighted gene co-expression network analysis (WGCNA), Mantini G and his colleagues constructed a prognostic signature. From their prognostic signature, they revealed that SPTBN1 could serve as an independent predictor of predicting OS of pancreatic ductal adenocarcinoma (PDAC) under clinical settings; they also found higher expression of SPTBN1 was associated with longer survival in patients with PDAC[50]. Moreover, Zhu H and his colleagues constructed a prognostic signature containing SPTBN1, and also found that SPTBN1 might be a potential tumor suppressor gene and could serve as an independent biomarker for predicting prognosis of patients with lung adenocarcinoma (LUAD)[51]. Similar to their findings, as shown in our study, we found higher expression of SPTBN1 was obviously correlated with longer survival in patients with KIRC, and could serve as an independent survival predictor for KIRC. Taken these results together, we speculate that SPTBN1 might have a potential to serve as a protective biofactor for some specific types of solid adenocarcinoma. A more comprehensive pan-cancer based on human solid adenocarcinoma is obviously needed to confirm this distinct biological manner of SPTBN1 in the future.

However, even though the bioinformatic analysis and following validation experiments in present study provided us some novel and reliable insights of SPTBN1 in clinical significance of KIRC and UVM, further molecular mechanistic experiments at cellular level and prospective clinical studies at multiple-center are both needed, which will be the focus of our future work.

## Conclusion

Overall, the findings from present study indicate that SPTBN1 could affect pan-cancer prognosis and closely associate with immune infiltration in TME. Specially, for KIRC and UVM, two different cancer types with different manners of aggressiveness, SPTBN1 upregulation is correlated with a low risk and immune infiltration for KIRC, and a high risk and immune infiltration for UVM. Importantly, to the best of our knowledge, there is no publication focusing on the prognostic and therapeutic roles of SPTBN1 in KIRC and UVM. Our data provide some new contents in this respect. By performing a series of bioinformatic analysis and following validation experiments, we demonstrated for the first time that SPTBN1 could be a potential prognostic and therapy-related biomarker in KIRC and UVM.

## Electronic supplementary material

Below is the link to the electronic supplementary material.

Supplementary Material 1

Supplementary Material 2

Supplementary Material 3

Supplementary Material 4

Supplementary Material 5

Supplementary Material 6

Supplementary Material 7

Supplementary Material 8

## Data Availability

The data used for bioinformatic analysis in this study have already been deposited in the cancer genome atlas (TCGA) database (https://portal.gdc.cancer.gov), clinical proteomic tumor analysis consortium (CPTAC) dataset (https://proteomic.datacommons.cancer.gov/pdc/) and gene expression omnibus (accession GSE44295) (https://www.ncbi.nlm.nih.gov/geo/query/acc.cgi?acc=GSE44295). The data used for external validation experiments in this study are available from the corresponding authors upon request via email.
