# Supplementary material for: Clinical significance of nonerythrocytic spectrin Beta 1 (SPTBN1) in human kidney renal clear cell carcinoma and uveal melanoma: a study based on Pan-Cancer Analysis
Source: BMC Cancer. 2023 Apr 3;23:303. doi: 10.1186/s12885-023-10789-3 (PMC10071745; doi:10.1186/s12885-023-10789-3)
Supplement: Supplementary file 1 — Supplementary Material 1 [file 12885_2023_10789_MOESM1_ESM.pdf]

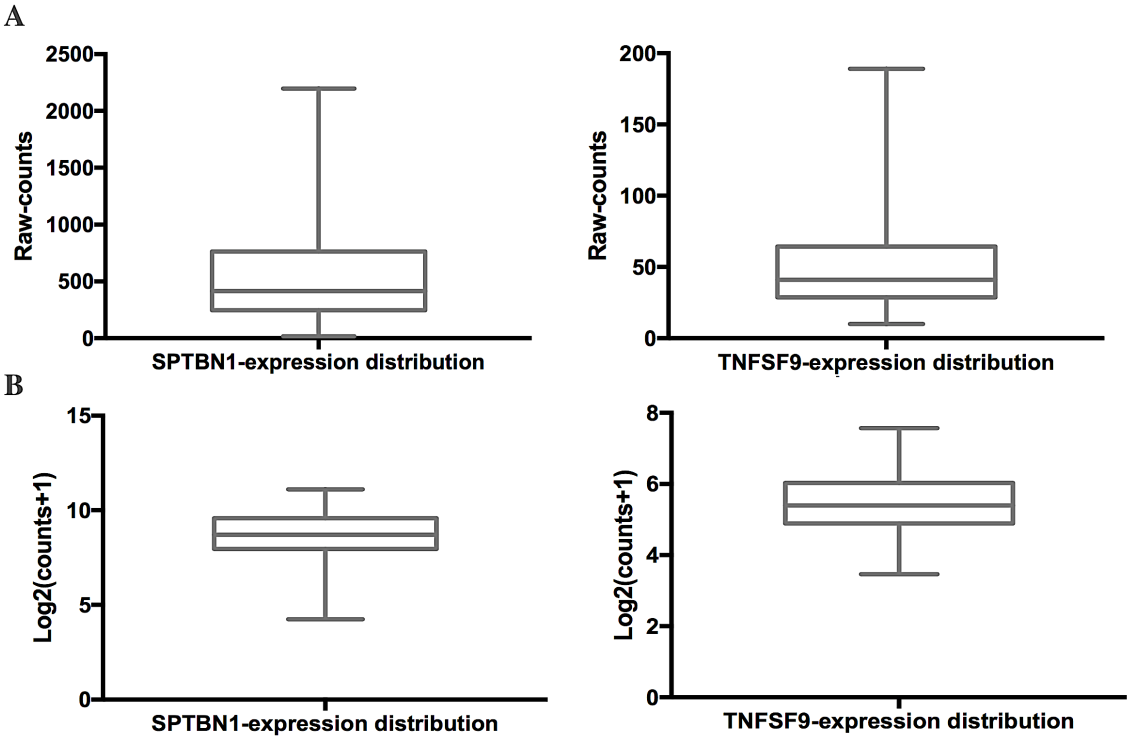

Supplementary File 1: The counts distributions of SPTBN1 and TNFSF9 expression obtained from GSE44295 dataset. **(A)** expression abundances in their raw-counts forms. **(B)** expression abundances in their log2(counts+1) normalized form. SPTBN1, non-erythrocytic spectrin beta 1; TNFSF9, tumor necrosis factor superfamily member 9.
