# Supplementary material for: Clinical significance of nonerythrocytic spectrin Beta 1 (SPTBN1) in human kidney renal clear cell carcinoma and uveal melanoma: a study based on Pan-Cancer Analysis
Source: BMC Cancer. 2023 Apr 3;23:303. doi: 10.1186/s12885-023-10789-3 (PMC10071745; doi:10.1186/s12885-023-10789-3)
Supplement: Supplementary file 2 — Supplementary Material 2 [file 12885_2023_10789_MOESM2_ESM.pdf]

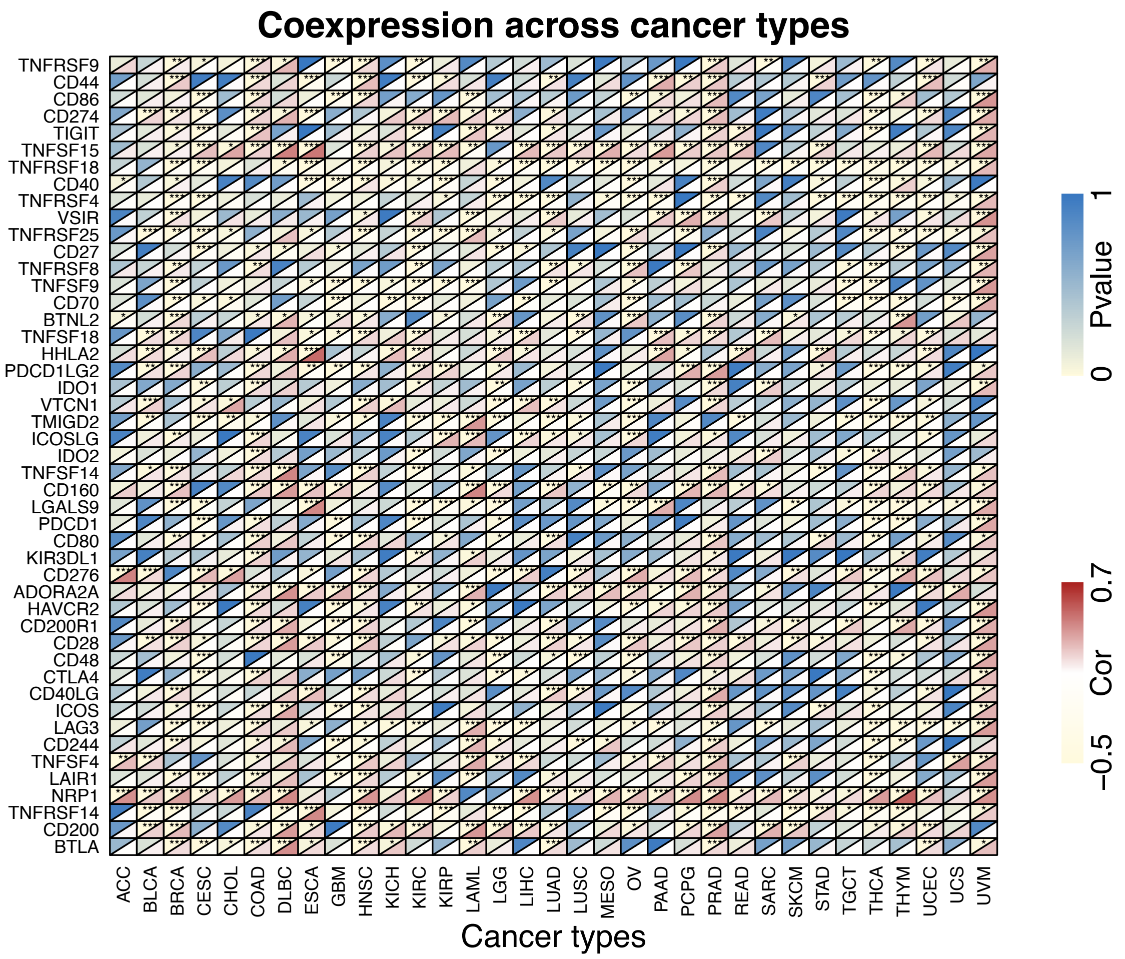

Supplementary File 2: Correlation between SPTBN1 expression and immunomodulator marker sets across TCGA cancers. * *P*<0.05; ** *P*<0.01; *** *P*<0.001. *P* value <0.05 were considered to be statistically significant.
